# Review on Magnetism in Catalysis: From Theory to PEMFC Applications of *3d* Metal Pt-Based Alloys

**DOI:** 10.3390/ijms232314768

**Published:** 2022-11-25

**Authors:** Chiara Biz, José Gracia, Mauro Fianchini

**Affiliations:** 1 MagnetoCat SL, General Polavieja 9 3I, 03012 Alicante, Spain; 2 Departamento de Química Inorgánica y Orgánica, Universitat Jaume I, Av. Vicente Sos Baynat s/n, 12071 Castellón de la Plana, Spain

**Keywords:** fuel cells, magnetism, ORR, magnetic catalysts, heterogeneous catalysis, clean energy

## Abstract

The relationship between magnetism and catalysis has been an important topic since the mid-20th century. At present time, the scientific community is well aware that a full comprehension of this relationship is required to face modern challenges, such as the need for clean energy technology. The successful use of (para-)magnetic materials has already been corroborated in catalytic processes, such as hydrogenation, Fenton reaction and ammonia synthesis. These catalysts typically contain transition metals from the first to the third row and are affected by the presence of an external magnetic field. Nowadays, it appears that the most promising approach to reach the goal of a more sustainable future is via ferromagnetic conducting catalysts containing open-shell metals (i.e., Fe, Co and Ni) with extra stabilization coming from the presence of an external magnetic field. However, understanding how intrinsic and extrinsic magnetic features are related to catalysis is still a complex task, especially when catalytic performances are improved by these magnetic phenomena. In the present review, we introduce the relationship between magnetism and catalysis and outline its importance in the production of clean energy, by describing the representative case of *3d* metal Pt-based alloys, which are extensively investigated and exploited in PEM fuel cells.

## 1. Introduction

The high pace of technological changes, the decarbonization of the power sector and climate change are just a few examples of the modern challenges that the world must address. Hydrogen technologies such as fuel cells have been identified worldwide as key enablers to help face these new challenges [1]. Proton exchange membrane fuel cells (PEMFCs) stand out among all designs of fuel cells as the most promising ones [2,3,4], particularly in the field of civil transportation [1,5,6,7]. However, several obstacles must be overcome in order to fully commercially exploit this technology [8,9]. From an electrochemical point of view, one such obstacle is the efficiency loss due to the overpotential of the oxygen reduction reaction (ORR), the most important catalytic step in the production of clean energy. The first catalyst historically employed in fuel cells was platinum (Pt) [10]. Pt is still the most employed material, despite its scarcity, nobility and high cost [1,10,11]. For these reasons, researchers over the past decade have mainly focused on finding optimal solid catalyst(s) with sufficient ORR activity, stability under operating conditions, an affordable price, wide availability and a small environmental footprint.

Magnetic catalysts based on *3d* metals (Cr, Mn, Fe, Co and Ni), such as bi-/trimetallic Pt-based materials, remarkably fit the desired profile [12]. The understanding of their outstanding catalytic properties entails the comprehension of complex chemical–physical phenomena related to the spins of the electrons. *3d*-transition metals (from Cr to Ni) and their alloys exhibit collective magnetism, a cooperative and spontaneous phenomenon among interacting electron spins [13]. Typical orderings of this collective behavior are ferromagnetism (FM), antiferromagnetism (AFM) and ferrimagnetism. Fe, Co and Ni metals display ferromagnetism, while Cr and Mn usually display antiferromagnetism [14,15].

Classical magnetostatic interactions cannot be the origin of this spontaneous and cooperative behavior. Dipole–dipole interactions, for instance, cannot explain the magnetic orderings found in real materials [15]. Thus, the origin of the cooperative behavior must be sought in a different class of interactions that are outside of the classical domain [15]. These are known as (indirect) exchange interactions, a quantum phenomenon with no classical analogue [16,17,18]. Indirect exchange interactions originate from the correlated movement of electrons with the same spin that allows an effective reduction of the electronic Coulomb repulsions [18]. These cooperative ferromagnetic spin electron interactions, together with spin-selective electron transport, represent some of the most important energetic contributions that enable milder chemisorption of reactants in heterogeneous catalysts [19]. Solid catalysts containing *3d* metals, such as Fe, Co and Ni, possess remarkable experimental ORR activity in fuel cells [11,20] and better catalytic performances in several other chemical transformations (e.g., water splitting reaction [21], Fischer–Tropsch process [22], hydrogen evolution reaction (HER) [23]). An outstanding example is the exploitation of a Pt/Co alloy as a PEM fuel cell catalyst in commercially available fuel cell electric vehicles (FCEVs) [5,24].

The present review is an improved evolution of the introductory section presented in the corresponding author’s doctoral thesis entitled “*Electronic and Magnetic Factors in the Design of Optimum Catalysts for Hydrogen Fuel Cells*” [25]. The aim of this current work is to provide a didactic introduction to the relationship between magnetism and catalysis. Given the multidisciplinarity, interdisciplinarity and the extent of the topic, the authors limit the treatment to the representative example of magnetic *3d* metal Pt-based alloys since they are exploited in commercial technologies for fuel cells. In order to provide a wide readership with an appropriate background, the readers are guided into the topic starting with a concise theoretical background on magnetism, magnetic materials and, especially, *3d* metals and their alloys (Section 2). The work continues by providing an overview on magnetism in catalysis, magnetic *3d* metal Pt-based alloys in oxygen oxidation reaction (ORR), and the enhancement of ORR by magnetism (Section 3). The attention is then focused on the basics of energy storage systems and fuel cells, particularly PEMFC devices, and modern strategies to exploit the relationship between catalysis and magnetism to enhance performances for the production of clean energy are emphasized (Section 4).

## 2. Magnetism in Transition Metals: The *3d*-Electrons Case

Magnetism is deeply interdependent with the concept of motion of elementary particles such as electrons (e.g., motion of charges and spin), whose spin is a quantum mechanical property [18,26]. The complexity arising from the interactions among moving charges and spins in solid-state matter can only be understood within the framework of quantum mechanics [13,15,26,27,28]. Magnetism has two main sources in solids: the *spin* and the *orbital magnetizations* [29]. The *spin magnetization* originates from the spin magnetic moment, while the *orbital magnetization* derives from the orbital magnetic moment. For a complete picture of magnetism in solids, both contributions have to be taken into account [29,30]. Despite this, the spin contribution is the most prominent one in a large variety of common materials, generally containing Fe, Co and Ni [29] (for this reason, the present work only focuses on *spin magnetization*). It is worth mentioning that the magnetic moments derived from nuclear spins also participate in the magnetization of a solid, but their contribution is generally neglected since the nuclear spin plays a minor role compared with the *spin* and *orbital magnetizations* [15,29].

### 2.1. Types of Magnetic Behavior

#### 2.1.1. Diamagnetism and Paramagnetism

The magnetic properties of a material at a macroscopic level can be identified according to its response to an applied external magnetic field ($\vec{H}$). A material becomes magnetized when it is subjected to a homogeneous external magnetic field ($\vec{H_{0}}+\vec{H}$, where $\vec{H_{0}}$ is the intrinsic magnetic field of the material in the absence of an external one). The measured quantity is called magnetization ($\vec{M}$), a specific property of each material [15,31], whose quantification is not a trivial task [15]. An experimentally more accessible parameter is instead the magnetic susceptibility of the material (χ) [15,32,33], from which it is possible to determine $\vec{M}$ (e.g., $\vec{M}=\chi \vec{H}$, valid for linear materials) [15,31]. Two fundamental types of magnetic behaviors can be identified depending on the sign of χ: diamagnetism and paramagnetism. A material is classified as diamagnetic when χ < 0 under the influence of an applied magnetic field ($\vec{H}$) [15,33]. Conversely, a material is classified as paramagnetic when χ > 0 under the influence of an applied magnetic field ($\vec{H}$) [15,33]. χ is usually independent from temperature in diamagnetic materials [13,15,33], whereas it is markedly temperature-dependent in paramagnetic materials [13,15,33].

**Figure 1:**
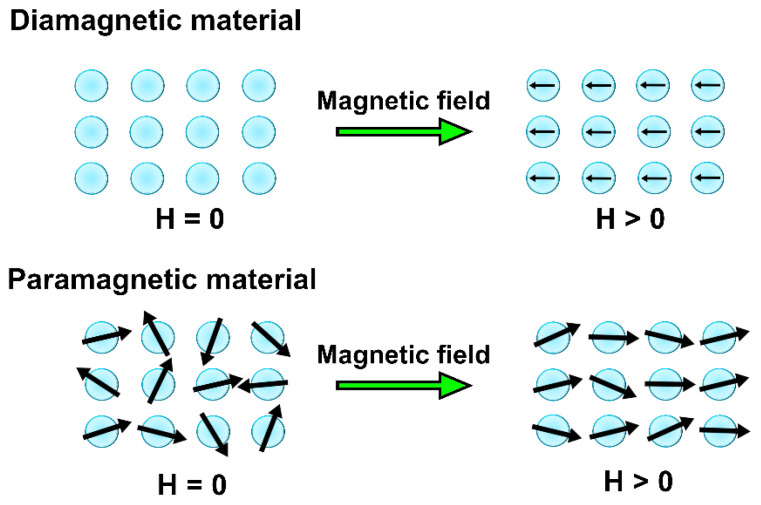
Simple sketch of the atomic magnetic moment configurations in diamagnetic and paramagnetic materials before and after the application of an external magnetic field ($\vec{H}$).

Diamagnetism is a property of all matter [13,15,33,34] and can be described as the magnetic response of electron configurations with fully filled orbitals shells (closed-shell configurations with paired electrons) towards an external magnetic field [31]: the field basically induces a perturbation into their orbital motion [34]. Diamagnetism is a weak phenomenon that can be observed only when other types of magnetism are completely inactive [15,34]. Figure 1 shows that the application of an external magnetic field to a diamagnetic material induces the magnetic moments to point in the opposite direction with respect to the direction of the external field [34]. Some examples of diamagnetic materials are metals such as mercury, copper and silver, as well as the majority of organic substances and most superconductors (below the critical temperature) [15,31].

Conversely, paramagnetism is the magnetic response of the interactions of the spin and/or orbital angular momenta (generally indicated as $\vec{S}$ and $\vec{L}$, respectively) belonging to open-shell configurations with unpaired electrons in the presence of an external field [15,33]. Under applied external field $\vec{H}$, the overall magnetization observed in paramagnetic materials is induced by the existence of oriented permanent magnetic moment [13,15,31], as seen in Figure 1. The origin of these permanent magnetic moments lies in the existence of a non-zero spin and orbital magnetic moments due to the stabilization and orientation of the unpaired electrons [15,34]. Figure 1 also shows that these permanent magnetic moments are randomly oriented when $\vec{H}\;$is absent [15,34]. Examples of paramagnetic materials are metals such as aluminum and sodium [14].

#### 2.1.2. Collective Magnetism

**Figure 2:**
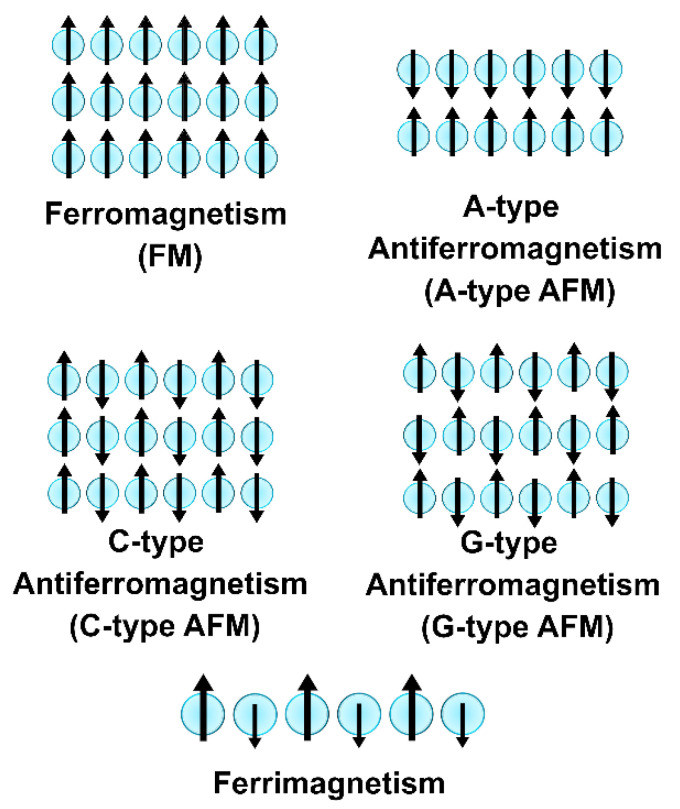
Scheme describing ferromagnetism, antiferromagnetism (most common types) and ferrimagnetism. The direction and the verse of the spins (atomic magnetic moments) are shown as black arrows. This is a simple scheme showing only one unpaired electron per atom, for clarity.

Some materials exhibit a spontaneous magnetization ($\vec{H_{0}}\neq 0$) even in absence of an external magnetic field ($\vec{H}=0$). The origin of this phenomenon is found in the correlated (cooperative) behaviour of interacting magnetic moments (spins) that promote a collective alignment/orientation in the electrons not subjected to any applied field. This particular magnetic phenomenon is called collective magnetism [13]. In materials displaying collective magnetism, adjacent magnetic centers can interact with each other through three possible interactions that also define their magnetic properties. The three types of behavior are called ferromagnetism, antiferromagnetism and ferrimagnetism (Figure 2).

The interactions among the magnetic moments of the electrons in a ferromagnetic (FM) material favor a parallel alignment between adjacent nearest atoms [15,33] that provide a net magnetization (i.e., spontaneous magnetization) to the material ($\vec{M}>0$) when no magnetic field is applied [13,15,34]. On the other hand, the material is called antiferromagnetic (AFM) when the magnetic moments of adjacent nearest atomic centers (or planes) with the same magnitude are coupled in an antiparallel fashion [15,33]. The total magnetization of an antiferromagnet is zero ($\vec{M}=0$) in the absence of an external field, due to the vectorial elimination of adjacent magnetic moments [13,15,31].

Various types of antiferromagnetism exist, but the most common types are A-type, C-type and G-type (Figure 2). A-type antiferromagnetism defines a situation where intra-plane coupling is ferromagnetic, while inter-plane coupling is antiferromagnetic. The opposite situation (i.e., intra-plane coupling is AFM and inter-plane is FM) defines C-type antiferromagnetism. Both intra- and inter-plane couplings are antiferromagnetic in the G-type antiferromagnetism. The third type of collective magnetism, ferrimagnetism, is defined by an antiparallel spin arrangement between adjacent magnetic moments having a dissimilar magnitude (Figure 2). Ferrimagnetism can also be described as two not-equal ferromagnetic sublattices coupled antiparallel with each other, whose magnetization is not canceled out [15,33]. Ferrites are a typical example of ferrimagnetic materials [15,31]. Figure 2 shows a schematic picture of the described examples of collective magnetism by using the collinear magnetic model (i.e., the coupling between two magnetic moments occurs at 0° or 180° with respect to each other). Ferromagnetic, antiferromagnetic and ferrimagnetic arrangements are also present in more complex configurations in real materials (e.g., helical order and spin glasses) [15], better described by the non-collinear magnetic model (i.e., the coupling between two magnetic moments occurs at different angles than 0° or 180° with respect to each other) [15].

The magnetic susceptibility (χ) is temperature-dependent in paramagnetic, ferromagnetic and antiferromagnetic materials. The temperature at which the susceptibility reaches its maximum is called Curie temperature (T_C_) and Néel point (T_N_) for ferromagnetic and antiferromagnetic compounds, respectively. T_C_ and T_N_ are specific for each material and indicate a change in the magnetic behavior of the compound [15]. The magnetic moments of these materials stop to behave “collectively” above the critical temperature and apparently start to act paramagnetically, following the well-known Curie–Weiss law [15,33].

#### 2.1.3. Strongly Correlated Electron Systems (SCES)

It is worth mentioning that correlated behavior of interacting magnetic moments can become intense in some materials due to strong electron–electron interactions. These magnetic compounds are known as strongly correlated electron systems (SCES) [28,35,36,37,38]. SCES are characterized by the simultaneous presence of various physical active interactions between the spins of the electrons, their charges, lattice and orbitals [28,36,37,38]. The simultaneous presence of several active physical interactions makes these systems attractive and suitable for device applications [38,39]. Complex transition metal oxides such as manganese oxides (i.e., manganites) are a paradigmatic example of such materials [28,37,38]. Strongly correlated materials represent a true challenge for experimentalists [28,38,39,40], as well as theoreticians [19,35,36,41,42,43], since they may display interesting phenomena such as colossal magneto-resistance effect, high-temperature superconductivity, multiferroic and magnetocaloric effects, metal–insulator transitions and negative thermal expansion [28,37,38,39,40]. Further effort is still needed to understand the properties and the behavior of such materials, particularly with the goal to exploit them in novel devices [28,39,43] and in heterogenous catalysts [19].

### 2.2. (Indirect) Exchange Interactions

Magnetic moments in classical physics, $\vec{\mu }$, are generated from electric current [15,41]. The interactions among these magnetic moments are called *dipole–dipole interactions*, magnetostatic interactions that depend on the distance between the two dipoles and on their relative orientation [41]. The Bohr–van Leeuwen theorem is valid in classical physics: the theorem states that “*in a classical system charges cannot flow in thermodynamic equilibrium*” [15,41]. This means that no magnetic moments should be observed in principle in any type of material (i.e., classically, their magnetization should be zero). However, the theorem is not experimentally validated [15,41], since a non-zero magnetization (i.e., spontaneous magnetization) is experimentally observed in many real materials, such as in ferromagnetic systems. Hence, classical physics cannot be used to explain the complex phenomenon of magnetism. Instead, according to quantum mechanics, current charges are common in ground states. The current density originates a magnetic moment that is proportional to the expectation value of electronic angular momentum ($\vec{L}$) and lies in the same direction, ${\vec{\mu }}_{L}=-\mu_{B}\langle \vec{L}\rangle$ (the proportionality constant $\mu_{B}$ is the *Bohr magneton*) [15,41]. Magnetic moments are also carried by the electron spins $\vec{(S})$, ${\vec{\mu }}_{S}=-g_{e}\mu_{B}\langle \vec{S}\rangle$ (the proportionality constant $g_{e}$ is the electron g-factor) [15,41]. Magnetic moments ($\mu =-e\hbar /2m_{e}$) in atoms have magnitude of $\mu_{B}$, the *Bohr magneton* ($\mu_{B}=e\hbar /2m_{e}$ = 9.274$\times 10^{-24}$ Am^2^) [15,33,41]. It is possible to estimate the value of the magnetostatic (direct) interaction of two magnetic moments μ separated by a distance of 1 Å as ~0.05 meV, which correspond to T < 1 K [41]. Yet, this cannot explain why magnetic orderings continue to exist at higher temperatures [41], such as intermetallic fct CoPt, which remains ferromagnetic at 750 K [14]. The consideration of magnetostatic interactions alone, however, cannot explain either the presence of spontaneous magnetism in some materials (i.e., long-range magnetic ordering) or the cooperative behavior observed in collective magnetic and in strongly correlated materials [15,28,41]. More complex interactions occur among magnetic moments: they are called (indirect) exchange interactions and have a quantum mechanical origin and no classical analogue [15,16,17,26].

#### 2.2.1. Basic Quantum Concepts

The main interest of quantum chemistry has been finding approximate solutions of the non-relativistic time-independent Schrödinger equation for a many-body system [18,26]. The Schrödinger equation for a system of N electrons and M nuclei defined by position vectors R_C_ and $r_{i}$, respectively, is described in Equation (1).

(1) $$\hat{H}|\Theta >=E|\Theta >$$

where $\hat{H}$ is the Hamiltonian operator, E is the eigenvalue corresponding to the energy level of the system and |Θ> is known as many-electron wave function and contains the entire information on the system.

The Hamiltonian $\hat{H}$ corresponds to Equation (2) [18,26,44]:

(2) $$\hat{H}=-\overset{N}{\underset{i=1}{\sum }}\frac{1}{2}\nabla_{i}^{2}-\overset{M}{\underset{C=1}{\sum }}\frac{1}{2M_{C}}\nabla_{C}^{2}-\overset{N}{\underset{i=1}{\sum }}\overset{M}{\underset{C=1}{\sum }}\frac{Z_{C}}{r_{iC}}+\overset{N}{\underset{i=1}{\sum }}\overset{N}{\underset{j>i}{\sum }}\frac{1}{r_{ij}}+\overset{M}{\underset{C=1}{\sum }}\overset{M}{\underset{D>C}{\sum }}\frac{Z_{C}Z_{D}}{R_{CD}}$$

where $Z_{C}$ and $Z_{D}$ are the atomic number of the nuclei *C* and *D*, $M_{C}$ is the ratio between the mass of *C* (nucleus) and an electron, $r_{iC}$ is the distance between the $i^{th}$ electron and the $C^{th}$ nucleus ($\left|r_{iC}\right|=\left|r_{i}-R_{C}\right|$), $r_{ij}$ is the distance between the $i^{th}$ and $j^{th}$ electrons ($\left|r_{ij}\right|=\left|r_{i}-R_{j}\right|$) and $R_{CD}$ is the distance between the $C^{th}$ and $D^{th}$ nuclei ($\left|R_{CD}\right|=\left|R_{C}-R_{D}\right|$). The Laplacian operators ($\nabla_{i}^{2}$, $\nabla_{C}^{2})$) are related to the spatial coordinates of the $i^{th}$ electron and the $C^{th}$ nucleus. The energy terms of $\hat{H}$ in Equation (2) are

- The operator for the kinetic energy of the electrons ($-\overset{N}{\underset{i=1}{\sum }}\frac{1}{2}\nabla_{i}^{2}$);
- The operator for the kinetic energy of the nuclei ($-\overset{M}{\underset{C=1}{\sum }}\frac{1}{2M_{C}}\nabla_{C}^{2}$);
- The electron–nucleus Coulomb attraction term ($-\overset{N}{\underset{i=1}{\sum }}\overset{M}{\underset{C=1}{\sum }}\frac{Z_{C}}{r_{iC}}$);
- The electron–electron Coulomb repulsion ($+\overset{N}{\underset{i=1}{\sum }}\overset{N}{\underset{j>i}{\sum }}\frac{1}{r_{ij}}$); and
- The nucleus–nucleus Coulomb repulsion ($+\overset{M}{\underset{C=1}{\sum }}\overset{M}{\underset{D>C}{\sum }}\frac{Z_{C}Z_{D}}{R_{CD}}$).

The Hamiltonian can be approximated by applying the *Born–Oppenheimer approximation*, since the nuclei are much heavier and move much slower than the electrons. This way, the obtained Hamiltonian describes the movement of N electrons in a field of M fixed nuclei (electronic Hamiltonian, Equation (3)) [18].

(3) $${\hat{H}}_{elec}=-\overset{N}{\underset{i=1}{\sum }}\frac{1}{2}\nabla_{i}^{2}-\overset{N}{\underset{i=1}{\sum }}\overset{M}{\underset{C=1}{\sum }}\frac{Z_{C}}{r_{iC}}+\overset{N}{\underset{i=1}{\sum }}\overset{N}{\underset{j>i}{\sum }}\frac{1}{r_{ij}}$$

${\hat{H}}_{elec}$ depends only on the spatial coordinates of the electrons, which do not entirely represent the properties of electrons. A complete treatment of electrons, in fact, is achieved only by identifying the electron spin or simply spin, a quantum mechanical property of these particles [18,26,44]. The spin consists of two orthonormal functions, conventionally indicated as spin up (↑ or spin α) and spin down (↓ or spin β), having a half-integral value 1/2 and −1/2 for fermions (electrons), respectively [18,26,44]. Consequently, an electron is fully described by its three spatial coordinates (r) and its one spin coordinate (ω), as x={r,ω}. A wave function Θ(x) can then be rewritten into two contributions: one depending on the spatial coordinates and the other depending on the spin; thus, Θ=ψ(r)·χ(ω) [18]. Similarly, it is possible to define a spin and a spatial orbital, since an orbital is a wave function for a single electron [18,26]. A spatial orbital $\psi_{i}\left(r\right)$ is the wave function of an electron that depends only on its position vector r, while a spin orbital (χ(x)) is the wave function of an electron fully described by its both spatial (r) and spin coordinate (ω) [18,26]. ${\left|\psi_{i}\left(r\right)\right|}^{2}dr$ describes the probability of finding an electron in a small volume dr around the point of r coordinates [18,26]. Two different spin orbitals can be formed from each spatial orbital $\psi_{i}\left(r\right)$, one for the spin up, α(ω), and the other for the spin down, β(ω). Spatial and spin orbitals are orthonormal [18,26].

The charge interactions among electrons, called electron–electron Coulomb interactions, are independent from their spin in the non-relativistic approximation (Equations (1) and (3)) [41,45] (as a matter of fact, there is no mention of the concept of spin in the expression of the Hamiltonian operator [18]). However, the total energy of a certain system (E) is also determined by its total spin, due to the principle of indistinguishability of similar particles (the principle states that two electrons described by the same spatial coordinates and spins are identical [26,27]) and the Pauli Exclusion principle [18,26,44].

The latter imposes the wave function (Θ) to be symmetric or antisymmetric [26,27,44]. The reason can be easily explained. If all the electrons are indistinguishable, they all must be treated the same way. So, when electrons are labeled in wave functions, their physical properties are independent from the label given to them. This non-preferential manner of labeling is called symmetrization [46]. Many-electron wave functions can be symmetrized in two ways. The first way entails the wave function being arranged in such a way that its sign does not change upon relabeling any two electrons (symmetric wave functions). The second way entails the wave function being arranged in such a way that its sign changes upon relabeling any two electrons (antisymmetric wave functions, i.e., two electrons cannot have the same quantum numbers). Both spatial and spin parts must be considered when taking into account the symmetry of many-electron wave functions towards the exchange of electron labels.

A wave function Θ (Θ=ψ(r)χ(ω)) of a system of N electrons with a half-integral spin (1/2,−1/2) must be anti-symmetric [18,26,27,44]. This means that if the spatial part of the function ψ(r) is symmetric, the spin part χ(ω).must be antisymmetric (i.e., the spins of two electrons must be antiparallel, ↑ and ↓). On the contrary, if the ψ(r) is antisymmetric, the spin part χ(ω) must be symmetric (e.g., the spin of two electrons are parallel, ↑ and ↑ or ↓ and ↓). In other words, the total wave function (ψ(r)+χ(ω)) must modify the sign if any two electrons are relabeled. Thus, the exact wave function must satisfy the Schrödinger equation and the so-called antisymmetry principle ($\Theta \left(x_{1},\ldots x_{i},\ldots x_{j},\ldots x_{N}\right)=-\Theta \left(x_{1},\ldots x_{j},\ldots x_{i},\ldots x_{N}\right)$) [18]. The antisymmetry principle is a general expression of the Pauli exclusion principle and states that “a many-body electron wave function must be antisymmetric with respect to the inter-change of the coordinate x (both spatial and spin) of any two electrons” [18]. As a consequence, the possible energy values for a system depend upon its total spin, that, in turn, arises from a quantum mechanical interaction called indirect exchange interaction [27].

#### 2.2.2. Exchange Effects

In order to understand how the requirement of antisymmetry is applied to the wave functions, we must start by considering a system of non-interacting *N* electrons. Such a system possesses the following Hamiltonian operator of Equation (4) [18]:

(4) $$\hat{H}=\overset{N}{\underset{i=1}{\sum }}h\left(i\right)$$

where h(i) represents the kinetic energy and the potential energy of electron i (the electron–electron repulsion is ignored). The h(i) operator possesses a set of eigenfunctions ($h\left(i\right)\chi_{j}\left(x_{i}\right)=\varepsilon_{i}\chi_{j}\left(x_{i}\right)$) that we can take as spin orbitals ($\chi_{j}$) [18]. Hence, the corresponding eigenfunction of the Hamiltonian ($\hat{H}$) (Equation (4)) is a wave function that is a product of spin orbital wave functions for every single electron ($\psi^{HP}\left(x_{1},x_{2},\ldots x_{N}\right)=\chi_{i}\left(x_{1}\right)\chi_{j}\left(x_{2}\right)\ldots \chi_{k}\left(x_{N}\right)$) [18]. $\psi^{HP}\left(x_{1},x_{2},\ldots x_{N}\right)$ is called *Hartree product*, a many-electron wave function where an electron i is described by its spin orbital ($\chi_{j}$) [18]. *Hartree product* is an independent-electron or uncorrelated wave function [18]. The probability of finding electron A at a specific point in space does not depend on the position of electron B. Postulating a system of non-interacting N electrons and $\hat{H}$ of Equation (4), something is missing in the *Hartree product*, precisely the notion of indistinguishability of electrons [18]. *Hartree product* distinguishes electron A occupying spin orbital $\chi_{i}$ and electron B occupying $\chi_{j}$ (and so on), but it does not satisfy the antisymmetric principle [18] that must be fulfilled in any wave function with half-integral spin. The issue can be solved by using the so-called *Slater determinant* [18,44], which enforces this prerequisite. For a system of N electrons, the *Slater determinant* corresponds to Equation (5):

(5) ψ(x1,x2,…xN)=(N!)−12|χi(x1)…χj(x1)………χi(xN)…χj(xN)|

The *Slater determinant* is fully defined by the occupied spin orbitals used to build it. The *Slater determinant* fulfills the Pauli exclusion principle in the sense that “*no more than one electron can occupy a spin orbital*” [18]. The most important effect of the antisymmetrization of the *Hartree product* to produce a *Slater determinant* is the introduction of *exchange effects*. These *exchange effects* arise from the condition of ${\left|\Psi \right|}^{2}$ (the square of the wave function is always positive) to be invariant with respect to the exchange of the spatial and spin coordinates of any two electrons [18]. Moreover, the *Slater determinant* includes the exchange correlation, according to which “*the motion of two electrons with parallel spins is correlated*” [18]. Thus, one may find the origin of the exchange correlation by investigating the effect of antisymmetrizing a *Hartree product*. The two-electron *Slater determinant* of $\chi_{A}$ and $\chi_{B}$ spin orbitals ($\Psi \left(x_{A},x_{B}\right)=|\chi_{A}\left(x_{A}\right)\chi_{B}\left(x_{B}\right)>$) can accommodate two electrons in two ways: opposite (antiparallel) spins or same (parallel) spins.

In the first case, antiparallel spins, the spin orbitals become as described in Equations (6) and (7):

(6) $$\chi_{A}\left(x_{A}\right)=\psi_{A}(r_{A})\alpha \left(\omega_{A}\right)\;$$

(7) $$\chi_{B}\left(x_{B}\right)=\psi_{B}(r_{B})\beta \left(\omega_{B}\right)$$

Therefore, one obtains the expression of the probability of simultaneously finding electron A in $dx_{A}$ and electron B in $dx_{B}$ by applying the determinant in Equation (8) [18]:

(8) $${\left|\Psi \right|}^{2}dx_{A}dx_{B}=\frac{1}{2}\;{\left|\psi_{A}(r_{A})\alpha \left(\omega_{A}\right)\psi_{B}(r_{B})\beta \left(\omega_{B}\right)-\psi_{A}(r_{B})\alpha \left(\omega_{B}\right)\psi_{B}(r_{A})\beta \left(\omega_{A}\right)\right|}^{2}dx_{A}dx_{B}$$

The probability of finding electron A in $dr_{A}$ at $r_{A}$ and electron B in $dr_{B}$ at $r_{B}$ at the same time can be indicated as $P\left(r_{A},r_{B}\right)dr_{A}dr_{B}$. This probability is calculated by integrating (averaging) the above Equation (8) over the spins of electron A and electron B (considering the principle of indistinguishability) [18].

(9) $$P\left(r_{A},r_{B}\right)dr_{A}dr_{B}=\int d\omega_{A}d\omega_{B}{\left|\Psi \right|}^{2}dr_{A}dr_{B}==\frac{1}{2}[{\left|\psi_{A}(r_{A})\right|}^{2}{\left|\psi_{B}(r_{B})\right|}^{2}+{\left|\psi_{A}(r_{B})\right|}^{2}{\left|\psi_{B}(r_{A})\right|}^{2}]dr_{A}dr_{B}$$

In Equation (9), the first term represents the product between the probability of finding electron A in $dr_{A}$ at $r_{A}$ and the probability of finding electron B in $dr_{B}$ at $r_{B}$, in the case that electron A and electron B occupy $\psi_{A}$ and $\psi_{B}$, respectively. The second term represents the same product, but in the case that electron A occupies $\psi_{B}$ and electron B occupies $\psi_{A}$. The overall probability corresponds to the average of these two terms since electrons are indistinguishable (Equation (9)). Therefore, the movement of the two electrons having opposite spins is uncorrelated [18]. This is clear if one considers $\psi_{A}=\psi_{B}$, since $P\left(r_{A},r_{B}\right)\;$becomes as in Equation (10) [18]:

(10) $$P\left(r_{A},r_{B}\right)={\left|\psi_{A}(r_{A})\right|}^{2}{\left|\psi_{A}(r_{B})\right|}^{2}$$

The meaning of Equation (10) is that two electrons having opposite spins can share the same spatial coordinates. On the contrary, when the two electrons possess the same α spin, the spin orbitals become as in Equations (11) and (12):

(11) $$\chi_{A}\left(x_{A}\right)=\psi_{A}(r_{A})\alpha \left(\omega_{A}\right)\;$$

(12) $$\chi_{B}\left(x_{B}\right)=\psi_{B}(r_{B})\alpha \left(\omega_{B}\right)\;$$

The new expression of $P\left(r_{A},r_{B}\right)\;$is described in Equation (13) [18]:

(13) $$\begin{array}{c} P\left(r_{A},r_{B}\right)=\frac{1}{2}\;[{\left|\psi_{A}(r_{A})\right|}^{2}{\left|\psi_{B}(r_{B})\right|}^{2}+{\left|\psi_{A}(r_{B})\right|}^{2}{\left|\psi_{B}(r_{A})\right|}^{2}-\\ -\left(\psi_{A}^{*}(r_{A}\right)\psi_{B}(r_{A})\psi_{B}^{*}(r_{B})\psi_{A}(r_{B})+\psi_{A}(r_{A})\psi_{B}^{*}(r_{A})\psi_{B}(r_{B})\psi_{A}^{*}(r_{B}))] \end{array}$$

The appearance of a third extra term (in bold) indicates that the probabilities are correlated, and it represents the exchange correlation between two electrons having the same spin [18]. In other words, when $\psi_{A}=\psi_{B}$, the probability $P\left(r_{A},r_{B}\right)$ is equal to zero, which means that two electrons with the same spin cannot occupy the same spatial–orbital. It is also said that a *Fermi hole* or an *exchange hole* exists around an electron under these conditions [18,47,48,49,50,51].

#### 2.2.3. Coulomb and Exchange Integrals

Since electrons are ubiquitous, the previous statements are valid for all diamagnetic and paramagnetic systems. This said, we can start the treatment from the definition of the ground state energy of a closed-shell system, where all the electrons are paired. An approximated ground state energy can be determined by applying the *Hartree–Fock method* [18,50]. The *Hartree–Fock approximation* essentially replaces the complex many-electron problem with a one-electron problem, and electron–electron repulsion is treated as an average quantity [18]. In addition, the N-electron wave function is approximated by a *Slater determinant* [52] that introduces exchange effects. Equation (14) shows the total Hartree–Fock energy ($E_{0}$) of a closed-shell ground state (the round brackets indicate that the sums involve the spatial orbitals), where a and b are the wave functions referred to as two electrons having different spins, and h comprises the average kinetic and nuclear attraction energy of an electron described by $\psi_{a}(r_{1}$) [18]:

(14) $$E_{0}=2\underset{a}{\sum }\left(a\left|h\right|a\right)+\underset{ab}{\sum }2\left(aa|bb\right)-\left(ab|ba\right)$$

The physical interpretation of Equation (14) is:

- (a|h|a) is a one-electron term (integral) that represents the average nuclear attraction and kinetic energy of an electron a described by $\psi_{a}(r_{1})$ (this integral is indicated as $h_{aa}$ in Equation (15)).
- (aa|bb) is a two-electron term (integral) that represents the classical Coulomb repulsion between ${\left|\psi_{a}(r_{1})\right|}^{2}$ and ${\left|\psi_{b}(r_{2})\right|}^{2}$ charge clouds, called *Coulomb integral* ($J_{ab}$).
- (ab|ba) is another two-electron integral with a quantum mechanics interpretation and it is called *exchange integral* ($K_{ab}$).

Coulomb ((aa|bb)) and the exchange integrals ((ab|ba)) possess positive values [18] and Equation (14) can be rewritten as follows [18]:

(15) $$E_{0}=2\underset{a}{\sum }h_{aa}+\underset{ab}{\sum }2J_{ab}-K_{ab}$$

It is worth reminding that the probability of finding two electrons having parallel spin (described by the wave function $|\bar{\psi_{1}}\;\bar{\psi_{2}}>$) at the same positions in space is zero, while the probability of finding two electrons with opposite spin (described by $|\psi_{1}\;\bar{\psi_{2}}>$) to share the same space is finite. Consequently, it is reasonable to assume that the energy of $|\bar{\psi_{1}}\;\bar{\psi_{2}}>$ (for example, E(↑↑)) is lower than the energy of $|\psi_{1}\;\bar{\psi_{2}}>$ (e.g., E(↑↓)), when the Coulomb repulsion between electrons is considered. Equations (16) and (17) express the energy of these two cases [18]:

(16) $$E\left(↑↑\right)=h_{11}+h_{22}+J_{12}-K_{12}$$

(17) $$E\left(↑↓\right)=h_{11}+h_{22}+J_{12}$$

The appearance of the stabilizing *exchange integral* ($K_{12}$) in Equation (16) makes E(↑↑)<E(↑↓). Its presence in a *Slater determinant* indicates that the motion of the electrons carrying parallel spin is correlated even in a single determinantal *Hartree–Fock* approximation of ψ [53]. The correlated motion of electrons with parallel spins reduces the electron repulsion, while the uncorrelated motion of electrons with antiparallel spins increases the Coulomb repulsion. This explains how the exchange effect, a consequence of the application of the antisymmetry principle, affects the possible energy values of a many-electron system. In addition, these concepts are at the basis of atomic multiplets and Hund’s first rule [13,41].

Following the same procedure, we can now define the total ground state energy for an open-shell system by applying the unrestricted form of the *Hartree–Fock approximation* [18], where spin α and spin β can be described by a different set of spatial orbitals ($\psi_{a}^{\alpha }\left(r\right)\neq \psi_{a}^{\beta }\left(r\right)$). By using the Coulomb and exchange integrals previously introduced, the ground state total energy for an open-shell system can be written as in Equation (18) [18]:

(18) $$E_{0}=\overset{N^{\alpha }}{\underset{a}{\sum }}h_{aa}^{\alpha }+\overset{N^{\beta }}{\underset{a}{\sum }}h_{aa}^{\beta }+\frac{1}{2}\overset{N^{\alpha }}{\underset{a}{\sum }}\overset{N^{\alpha }}{\underset{b}{\sum }}\left(J_{ab}^{\alpha \alpha }-K_{ab}^{\alpha \alpha }\right)+\frac{1}{2}\overset{N^{\beta }}{\underset{a}{\sum }}\overset{N^{\beta }}{\underset{b}{\sum }}\left(J_{ab}^{\beta \beta }-K_{ab}^{\beta \beta }\right)+\overset{N^{\alpha }}{\underset{a}{\sum }}\overset{N^{\beta }}{\underset{b}{\sum }}J_{ab}^{\alpha \beta }$$

where:

- $h_{aa}^{\alpha }=\left(\psi_{a}^{\alpha }|h|\psi_{a}^{\alpha }\right)$ and $h_{aa}^{\beta }=\left(\psi_{a}^{\beta }|h|\psi_{a}^{\beta }\right)$ are the averages of the nuclear attraction and kinetic energy for an electron a described by $\psi_{a}^{\alpha }\left(r\right)$ and$\;\psi_{a}^{\beta }\left(r\right)$;
- $J_{ab}^{\alpha \alpha }=\left(\psi_{a}^{\alpha }\left|J_{b}^{\alpha }\right|\psi_{a}^{\alpha }\right)=\left(\psi_{b}^{\alpha }\left|J_{a}^{\alpha }\right|\psi_{b}^{\alpha }\right)$ and $J_{ab}^{\beta \beta }=\left(\psi_{a}^{\beta }\left|J_{b}^{\beta }\right|\psi_{a}^{\beta }\right)=\left(\psi_{a}^{\beta }\left|J_{b}^{\beta }\right|\psi_{a}^{\beta }\right)$ are the *Coulomb integrals* between electrons with the same spin,
- $J_{ab}^{\alpha \beta }=J_{ab}^{\beta \alpha }=\left(\psi_{a}^{\alpha }\left|J_{b}^{\beta }\right|\psi_{a}^{\alpha }\right)=\left(\psi_{a}^{\beta }\left|J_{b}^{\alpha }\right|\psi_{a}^{\beta }\right)$ is the *Coulomb integral* of an electron in $\psi_{a}^{a}$ with one in $\psi_{b}^{\beta }$;
- $K_{ab}^{\alpha \alpha }=\left(\psi_{a}^{\alpha }\left|K_{b}^{\alpha }\right|\psi_{a}^{\alpha }\right)=\left(\psi_{b}^{\alpha }\left|K_{a}^{\alpha }\right|\psi_{b}^{\alpha }\right)$ and $K_{ab}^{\beta \beta }=\left(\psi_{a}^{\beta }\left|K_{b}^{\beta }\right|\psi_{a}^{\beta }\right)=\left(\psi_{b}^{\beta }\left|K_{a}^{\beta }\right|\psi_{b}^{\beta }\right)$ are the *exchange integrals* among electrons with parallel spin (there is no exchange interaction between electrons with antiparallel spin);
- The summations, with upper limit $N^{\alpha }$ and $N^{\beta }$, are over occupied orbitals $\psi_{a}^{\alpha }$ or $\psi_{b}^{\alpha }$ and $\psi_{a}^{\beta }$ or $\psi_{b}^{\beta }$, respectively; and
- The factor 1/2 in the third and fourth terms removes the double counting in the free sum.

The electron–electron interactions in an open-shell system are more diversified than in a closed-shell one (Equation (14)), as seen in Equation (18). Spin α perceives a Coulomb potential ($J_{ab}^{\alpha \alpha }$) and an exchange one ($K_{ab}^{\alpha \alpha }$) from each $N^{\alpha }$ electron of same spin α occupying $\psi_{a}^{\alpha }$, plus a Coulomb potential $J_{ab}^{\alpha \beta }$ from each $N^{\beta }=N-N^{\alpha }$ electron of opposite spin β occupying $\psi_{a}^{\beta }$ [18]—this represents the effective potential observed by an electron with spin α in an open-shell system. The same conclusions are also valid for electrons with spin β. It is possible to define now *an (indirect) exchange interaction as an interaction originated from the correlated movement of electrons with the same spin* [15,16,17,53]. Such interaction includes the scattering mechanism between electrons with parallel spins, allowing an effective reduction of the electronic Coulomb repulsion [16,54].

**Figure 3:**
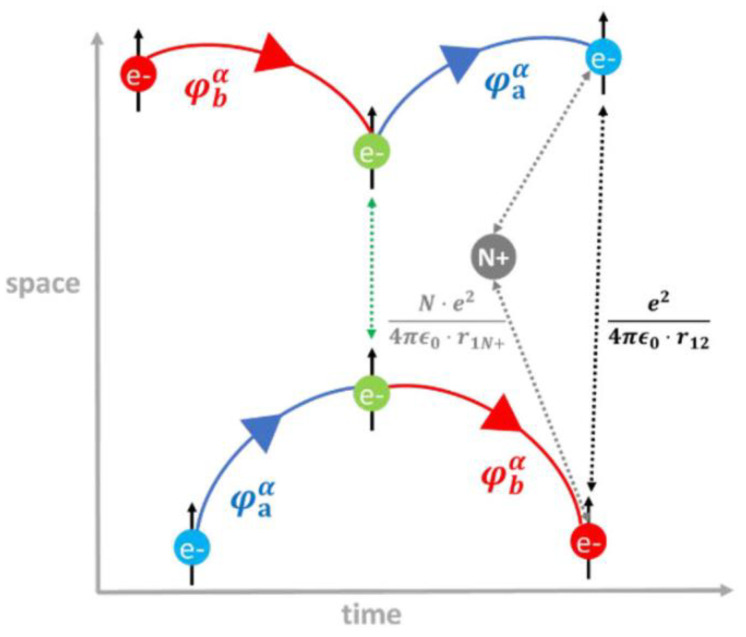
Feynman-type diagram (space (X) vs. time (Y)) of a quantum spin exchange interaction (QSEI) between two electrons with the same spin (α) in $\varphi_{a}^{\alpha }$ and $\varphi_{b}^{\alpha }$ orbitals. The curved lines define the space–time evolution of the electrons in $\varphi_{a}^{\alpha }$ and $\varphi_{b}^{\alpha }$, under the influence of the repulsive quantized Coulomb potentials formed between the electrons. The direction of the space (y)–time (x) development is indicated by the arrow [19,54]. Adapted with permission from Biz, C.; Fianchini, M.; Gracia, J. Strongly Correlated Electrons in Catalysis: Focus on Quantum Exchange. *ACS Catalysis* 2021, 11 (22), 14249–14261. Copyright 2021, American Chemical Society.

Feynman diagrams, commonly used to visualize particle trajectories [44,54,55], are also helpful in portraying graphically an (indirect) exchange interaction. Figure 3 displays a Feynman-type space–time representation of the interaction between two electrons with the same spin (α in this case) residing in two different orbitals ($\varphi_{a}^{\alpha }$ and $\varphi_{b}^{\alpha }$). Such an interaction involves the presence of an additional scattering matrix, the exchange integrals of Equation (18), that can be expressed as $-\langle \varphi_{a}^{\alpha \left(\beta \right)}\varphi_{b}^{\alpha \left(\beta \right)}|\frac{e^{2}}{4\pi \epsilon_{0}\cdot r_{12}}|\varphi_{a}^{\alpha \left(\beta \right)}\varphi_{b}^{\alpha \left(\beta \right)}\rangle$ for two electrons with the same spin occupying two different orbitals. The operator $(\frac{e^{2}}{4\pi \epsilon_{0}\cdot r_{12}}$) is the electron–electron Coulomb repulsion [18]. Through the concept of exchange integrals, quantum mechanics introduces the possibility that two indistinguishable electrons with parallel spins can exchange their orbitals, position and momentum [54]. Again, these are quantum phenomena that arise from the imposition of the antisymmetric principle to the electron wave function, under the Pauli exclusion principle [27,53]. In light of all the concepts previously discussed, some authors called (indirect) exchange interactions quantum spin exchange interactions (QSEIs) in the field of heterogeneous catalysis, in order to underline their nonclassical origin and the involvement of the spin electron component in the catalytic process [19,54,56]. The same authors proposed the concept of quantum excitation interactions (QEXIs) to describe electron–electron scattering involving excited states [19].

#### 2.2.4. Exchange Mechanisms

Effective coupling between spins (i.e., magnetic moments) is established in solids via the interplay of the electron–electron Coulomb repulsion (Coulomb exchange) and the hopping of electrons from one orbital to another one belonging to a neighboring atom (kinetic exchange) [28], provided that the Pauli exclusion principle results are always satisfied [41]. The kinetic exchange (e.g., actual movement of the charge carriers) is also regulated by Coulomb repulsion between the electrons—this means that the hopping to an orbital of a neighboring atom can occur only if that orbital is not already occupied by an electron with the same spin [41]. In other words, the hopping processes are only allowed if the orbital is occupied by electrons with antiparallel spins in the neighboring atom [28]. The strength of such hopping depends on the interatomic separation [27] and it allows a further lowering of the Coulomb repulsion [28]. The realistic coupling mechanisms acting among the electron spins in real materials are complex, but simplifications are possible by introducing model mechanisms called exchange mechanisms. The types of exchange mechanisms are *direct exchange*, RKKY, *anisotropic* exchange interaction, *super-exchange* and *double-exchange*.

**Figure 4:**
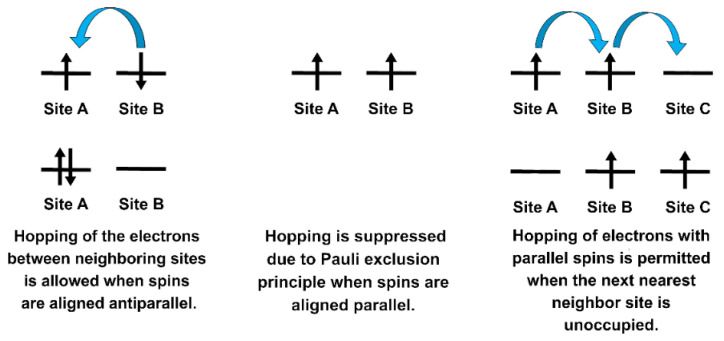
Simple sketch of the direct exchange mechanism between antiparallel (**left**) and parallel spins (**right**).

Under the *direct exchange mechanism* (Figure 4), the orbitals of two sites are close enough to allow a viable overlapping of their lobes; as a consequence, a direct electron hopping occurs between neighboring magnetic centers [15,28,41]. Despite its inherent “simplicity”, this is a short-range mechanism, and examples of direct exchange are hardly found in real materials [41].

The RKKY (Ruderman, Kittel, Kasuya, Yosida) exchange interaction [57,58,59] (or *indirect exchange* or *itinerant exchange*) involves the coupling between spins located at relatively large distances (i.e., there is no direct overlap between neighboring orbitals) through the mediation of electrons having an itinerant character (i.e., conduction electrons, see next paragraph). These electrons spread the induced spin polarization over the neighborhood [13,15]. The interaction is a long-range one and depends on the distance between the magnetic centers (it can be either AFM or FM) [15]. Thus, indirect exchange creates magnetic orderings [36]. RKKY interaction is an important exchange mechanism in metals. For example, when metals such as Cu are doped with *3d* magnetic metals or rare earth elements, the interactions established between the *d* or *f* electrons of the impurities, and the conduction electrons are RKKY interactions [60]. The same interactions are also used to explain the transmission of spin polarization among magnetic/nonmagnetic multilayers [31,61,62].

Another important exchange mechanism is the *anisotropic exchange interaction* or *Dzyaloshinsky–Moriya interaction* [15,63,64], in which the spin–orbit coupling is involved in the exchange mechanism in a similar way to that of oxygen atom in the *super-exchange* mechanism. The *spin–orbit coupling* in an atom arises from the interaction between the spin and the orbital component of the wave function of the electron [15,28,33].

In the *super-exchange* and *double-exchange mechanisms*, the electron hopping between two non-neighboring magnetic centers (M) is assisted by intermediary orbitals of a nonmagnetic atom (usually *p*-orbitals of an oxygen atom) [15,28,41,65]. In a super-exchange mechanism, the exchange on the connecting atom is regulated by the Coulomb exchange (i.e., the occupation of the M orbitals) and the angle between the two magnetic centers and the nonmagnetic atom [28]. In a *double-exchange mechanism*, both Coulomb and kinetic exchange work in a combined fashion [41]. Established rules exist to help distinguishing between *super-exchange* and *double-exchange* cases, known as Goodenough–Kanamori rules [27,28,65,66]. Examples of *super-exchange* and *double-exchange* are widely common in materials: *super-exchange interactions* are found in ionic solids such as perovskites [13,28,65], while double-exchange interactions are found in compounds having mixed-valence metal centers (e.g., manganites and magnetite-Fe_3_O_4_) [15,28,41].

### 2.3. Electronic Structure of Solids

A solid is described by a crystalline structure, characterized by a regular arrangement of its components (atoms, ions, molecules) into a fixed and rigid periodically repeated pattern called crystal lattice [67,68]. A space lattice is defined as a regular and homogeneous arrangement of discrete points (lattice points) in the three dimensions (lattice vectors) [67]. In order to investigate the properties of these 3D periodic arrays, the identification of their smallest repeated unit, called the unit cell (reflecting the whole symmetry of the crystal structure), is pivotal [67,68,69]. All the elements constituting crystalline structure can be simplified into two main classes: the atomic cores, also called ions (nuclei + core electrons), and the electrons outside of the core (the valence electrons) [32,68]. The comprehension of the electronic structure in solids and the connection between electronic and physical properties is a challenging task. Typically, three models are used: the free electron Fermi gas (or Sommerfeld model), the Fermi liquid and the band structure models. The first one was proposed as the first model to achieve this goal [32]. According to this model, a metal is treated as a 3D potential box, where the electrons can move “freely” (obeying Pauli exclusion principle) and the Coulomb electron–electron interactions are not explicitly taken into account [15,28,32]. Such electron–electron interactions are, instead, considered in the Fermi liquid model as a first approximation through the introduction of quasi-particles. In this second proposed model, the electrons not only have a charge and a spin (as stated in the free electron model), but also possess an effective mass (dressed electrons) [28,32]. The third model is the band structure model, which provides further improvements by including the presence of a periodic potential [28,32], as elucidated in the next paragraph. It allows a successful description of several properties of solids [28].

#### 2.3.1. Bloch Theorem

As for the atomic case, the many-electron problem in solids is treated like a single electron moving in a potential (V(r)) generated by the average behavior of the nuclei and the remaining electrons [27,28]. This single electron treatment includes some relevant concepts such as the possibility to treat the electron outside of the core as one-electron system and to describe them by using solutions of a single electron moving in a periodic potential [27].

The physicist Felix Bloch found that in a solid (that usually is a 3D periodic array), this electronic potential V(r) generated by the atomic cores (or ions) [27,32] possesses the periodicity of the crystal structure. Such periodicity can be described by a lattice vector T [70]. Consequently, the electrons in a solid are subjected to a periodic potential extended in three dimensions V(r+T)≡V (r) [70]. One can generate extended wave functions for a crystal, also known as electronic bands, by applying this 3D translational invariance of the crystalline solid and Bloch theorem.

The Bloch theorem states that “for a given wave function ψ(κ, r), that fulfills the Schrödinger equation, a vector κ exists such that translation by a lattice vector T is equivalent to multiplication by a phase factor” [26,44,70], mathematically expressed by Equation (19).

(19) $$\psi \left(\kappa ,r+T\right)=e^{i\kappa T}\psi \left(k,r\right)$$

ψ(κ,r+T) is an extended wave function called a Bloch wave function (lattice periodic function), also indicated as $\psi_{k}(r$), which depends on a lattice vector T and on the quantum number κ, while ψ(κ, r) is a wave function depending only on κ [32]. The meaning of the Bloch theorem is that any possible solution of the Schrödinger equation ψ(κ, r) differs between equivalent positions in the solid structure only by a factor of $e^{i\kappa T}$. Equation (19) also explains that electrons perceiving a periodic potential could remain delocalized (nearly free) [71]. The extended wave function ψ(κ,r+T) in the Bloch theorem is generated at any specific point inside the crystal having r spatial coordinates, and the combination r+T corresponds to the whole crystal structure. κ is a quantum number, introduced by the Bloch theorem, that possesses the dimension of an inverse length, and thus κ is an occupant of the so-called reciprocal space (or κ-space). More precisely, the reciprocal space is restricted to a specific space interval known as the Brillouin zone [69,70]. The first Brillouin zone [27,69] is the unit cell in the reciprocal space. The reciprocal space reflects the periodicity of the space coordinates of the real space of a crystal by a unique reciprocal lattice [27,32,69]. Real and reciprocal spaces are mathematically related [70]. The generated crystal wave function (or crystal orbital) ψ(κ, r) is symmetry-adapted to the periodic system thanks to κ [70]. Infinite independent solutions of the Schrödinger equation exist for a specified value of κ: these solutions vary among one another by an energy quantum number, known as the band index (n) [32]. The energy eigenvalues for all n and κ values describe the energy band structure of the electrons in a periodic potential (E(ψ(k,r)) or $E_{k}$) [28,32,70]. One electron of either spin per unit cell can be accommodated in each energy band [32,70]. Moreover, E(ψ(k,r)) is subjected to the translational invariance of the crystalline solid structure, ($E_{n}\left(k,\;r\right)$ = $E_{n}\left(k,\;r+T\right)$), so that the energy band structure completely reproduces the symmetry of the crystal [32].

Some important concepts are connected to the Bloch theorem, for example, *Bragg reflection* and *Fermi energy*.

- The *Bragg reflection* is a feature of the wave propagation in periodic systems; hence, it must be also a characteristic of electron waves in crystalline structures [27,32,69]. The most relevant consequence of the *Bragg reflection* is that it leads to the creation of gaps in the distribution of energy states [27]. The energy spectrum of a crystal is transformed into a band structure featuring energy levels where the propagation of electrons becomes possible [32]. The Bragg reflection conditions can also be used to build the boundaries of the Brillouin zone [27].
- The *Fermi energy* (E_F_) is a concept of quantum mechanics used in solids. The Fermi energy defines the energy level for which all states having energy € smaller than E_F_ are occupied at T = 0 K [15,26,32]. In other words, E_F_ represents the highest occupied energy level [13,44]. There are no thermal energies at 0 K, so the occupation of one-electron states is determined only by placing one electron per state (in agreement with the Pauli exclusion principle). The position of E_F_ in relation to the band energy level is important in determining the electronic and thermal properties of a solid, since it energetically separates the occupied from the non-occupied states [32,44]. Another useful concept related to *Fermi energy* is the *Fermi surface*. The *Fermi surface* is a surface, defined in the reciprocal space, that separates the occupied states from the empty ones at 0 K [32,71]. Its shape can provide information on the electrical properties of a solid [71]. The electronic bands placed below and above E_F_ are called valence and conduction bands, respectively [32].

**Figure 5:**
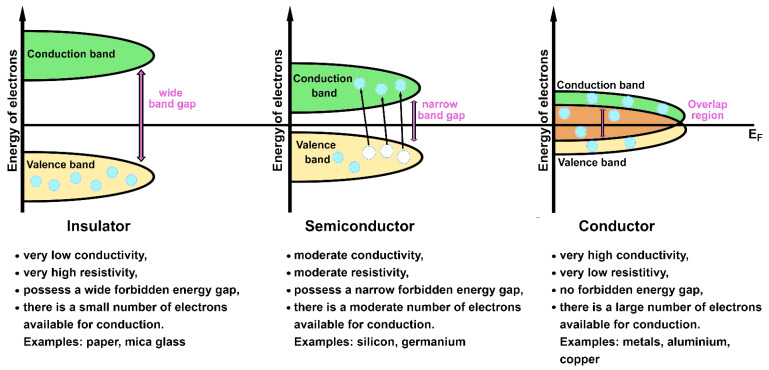
Simple sketch of the differences between insulators, semiconductors and conductors.

The classification of solids in *conductors*, *semiconductors* and *insulators* arises from the observations that electrons occupying filled bands do not carry any electric current (i.e., not all the solids are metals) [27,71] and that energy gaps are enclosed at the Brillouin zone boundary (as a consequence of the combination between Bragg reflection and Bloch theorem) [71]. A crystal with partially filled conduction and valence bands is defined as *metallic conductor*; a characteristic overlap between the valence and conduction bands, containing E_F_, is shown by metals (i.e., no forbidden energy gap is present). The solids with filled valence bands are considered *insulators*; there is a wide energy gap between the valence and the conductions bands in *insulators*. When the energy separation between the valence and the conduction bands is narrow (comparable to $k_{b}T$, where $k_{b}$ is the Boltzmann constant), the material is classified as a *semiconductor*, a subgroup of *insulators* [28,32,71]. A large amount of external energy (e.g., provided by the increment of temperature) must be applied to *insulators* to move the electrons from the filled valence band to the empty conduction one. The opposite situation is valid for *conductors*, where electrons already occupy the conduction band (that results somewhat filled) like the valence one at room temperature. In a *conductor material*, electrons can move “freely” in the overlap region by carrying an electric current. *Semiconductors* show an intermediate behavior: they behave as insulators at low temperatures and as conductors at room and higher temperatures. Some electrons can acquire the necessary energy to overcome the narrow energy gap and reach the conduction band in semiconductors upon application of an external source (e.g., thermal energy, light and so on). Schematic representations of these three types of materials and their characteristics are shown in Figure 5.

#### 2.3.2. Electrons in Transition Metals

The first model used to treat electrons in transition metals is called the *itinerant electron model* [72,73,74]. According to this model, the electrons are assumed to move “freely” within the solid structure [33] and are responsible for the metallic conductivity of the material [72] (assumption used in the band structure model [45]). The opposite situation is when such electrons are localized (*localized electron model*) [33,73] and are considered to carry a non-conducting character [72] (assumption considered in the crystal-field theory [15,33,45]). For example, *4f*-electrons in rare earth metals and *5f*-electrons in actinides are generally described as localized [13,32], while *d*-electrons (especially *3d*-electrons) in transition metals are commonly considered as itinerant [72,74]. The two models can coexist [27]. The electronic structure in real materials, however, is generally more complex than these two simple representations.

The itinerant electron model is based on Bloch wave functions ψ(κ,r+T) or $\psi_{k}\left(r\right)$ (Equation (19)) that are solutions of a one-electron Schrödinger equation [72] (see Equation (20)).

(20) $$\left(-\frac{\hbar^{2}}{2m}\nabla^{2}-V\right)\psi_{k}\left(r\right)=E_{k}\psi_{k}\left(r\right)$$

where V is the periodic potential. The corresponding approximate many-electron wave function (Ψ) for the whole system can be written by using the *Slater determinant* under the *Hartree–Fock approximation* [72]. The *Slater determinant* is built from $\psi_{k}\left(r\right)$ of occupied states (the energy states below the *Fermi energy*). The application of the *Slater determinant* introduces the exchange effects, as already described, but it does not take into account all the effects related to the electron–electron interactions, specifically the correlation among the positions of electrons with opposite spin [72]. Indeed, the electronic correlations are only loosely approximated in the previous models used to described electrons in solids (i.e., the *free electron Fermi gas*, *Fermi liquid* and *band structure models*) [28]. A possible solution is to reduce the periodic potential of the electron–electron correlation to a small pseudopotential [72] treatable by perturbational theory [72,75]. However, such treatment is not possible for *d*-electrons of transition metals [72]. More suitable methods must be used to describe the electron–electron interactions and the electron correlation. This is especially true for *3d*-transition metals, where the correlated behavior of the outer *3d*-electrons promotes a collective alignment of their spins (collective magnetism). The electrons responsible for the electronic and magnetic properties in *3d*-transition metals and their alloys are the *3d*-valence electrons (outside of the core) [27,32,74]. The exception is rare earth metals whose magnetic properties arise from the inner *4f*-shells [27,60,76].

Two important models are commonly used to describe interacting electrons: the *Heisenberg model* and the *Hubbard model*. These models employ different approaches to solve the electron–electron interaction and the electron correlation. Moreover, these models, together with others, such as the *Stoner model* [13,15,32], have been formulated to address the topic on the origin of ferromagnetism in *3d*-transtion metals [77]. Indeed, it appears that ferromagnetism arises from electron–electron interactions [28]. However, the majority of the interactions among electrons in solids are Coulomb interactions that are completely spin-independent [27,78]. This discrepancy rises a fundamental issue: “Can spin-independent interactions be the origin of ferromagnetic ordering in a collective electronic system?” Answering this question is not only fundamental to understand the ferromagnetic phenomenon, but also to comprehend the role of non-linear interactions in many-electron systems [78].

#### 2.3.3. Heisenberg Model

Heisenberg proposed a model of interacting electrons in 1928 to quantomechanically explain the origin of ferromagnetism in iron (Fe), cobalt (Co) and nickel (Ni) [27,31]. The model assumes that the electrons have a localized character and a full electron correlation [15,70]. It considers local interactions, thus the interactions among nearest neighbor atomic magnetic moments that align them when no external magnetic field is applied [31]. The effective spin interaction between two orbits $\Phi_{i}$ and $\Phi_{j}$ having $S_{i}$ and $S_{j}$ spin angular momenta is described as a perturbative potential energy of Equation (21) [27] in the *Heisenberg model*.

(21) $$V_{ij}=-2J_{ij}S_{i}S_{j}$$

where $J_{ij}$ corresponds to the effective exchange integral between atoms i and j [13,27] (previously indicated as $K_{ab}$, Equation (15)). The sign of the exchange integral indicates if the coupling between the two neighboring atoms is ferromagnetic (i and j possess parallel spin alignment) or antiferromagnetic (i and j possess antiparallel spin alignment) [13,15,28,32]. Thus, positive $J_{ij}$ values are associated with FM couplings since the triplet state is stabilized for $J_{ij}>0$, while negative $J_{ij}$ values are related with AFM couplings, since, in this case, the singlet state is stabilized for $J_{ij}<0$. Equation (21) can be generalized for a many-electron system as *Heisenberg exchange Hamiltonian* [13,15,28,32,79] (Equation (22)).

(22) $$H_{spin}=-\underset{ij}{\sum }J_{ij}S_{i}S_{j}$$

The factor 2 is omitted from Equation (22), indicating that the sum includes each pair of spins twice [15,27,41]. Due to its “simplicity”, the *Heisenberg model* does not work properly for systems characterized by indirect coupling mediated by *s*-*p* electrons, as in rare earth metals, in dilute transition metal alloys [27] and in correlated materials [28]. The *Heisenberg model* does not perform well in all those cases where the magnetic behavior of an atom does not depend on the magnetic one of its neighborhood [33]. This is a consequence of $J_{ij}$ sensitivity to orbital overlap [27]. Some authors also pointed out that the application of the *Heisenberg model* is not rigorous for many-electron systems, due to a “*lack of orthogonality of the one-electron orbitals of atomic type*” [80]. Moreover, even though the model was formulated to explain the ferromagnetic properties of Fe, Co and Ni, it treats the outer *d*-electrons of these transition metals as localized when they actually possess a strongly delocalized character (itinerant electrons) [70]. The itinerant character of these electrons is confirmed by the experimental determination of their non-integer magnetic moments: 2.22 $\mu_{B}$, 1.715 $\mu_{B}$ and 0.606 $\mu_{B}$ for bcc Fe, hcp Co and fcc Ni, respectively [15,81]. The *Heisenberg model* also disregards aspects of the electron–electron interactions that should not be neglected [80]. Despite all these criticisms, the *Heisenberg model* represents a good starting point to understand the magnetic properties of magnetic materials.

#### 2.3.4. Hubbard Model

More articulated approaches than the *Heisenberg model* have been introduced throughout the years [27,28]. One of these models is the *Hubbard model*. It contains the main aspects to describe interactions among quantum mechanical particles (such as electrons) moving in a periodic potential. It is also known as “lattice Fermion model”. The model was proposed independently by various scientists to solve different tasks, such as the description of transition metals (J. Hubbard), the description of the itinerant ferromagnetism (J. Kanamori) and the characterization of the metal–insulator transitions (M. C. Gutzwiller) [82]. The *Hubbard model* is based on a tight binding approach [15,28,82]; in other words, it assumes that atoms in a solid-state material are involved in almost no chemical interaction [70].

Its basic idea is that only one (or a few) energy band near the *Fermi energy* contributes to define the ground state energy of the system [70]. The corresponding Hamiltonian is presented in Equation (23).

(23) $$H=H_{kin}+H_{int}=\underset{x,y\in V,\sigma }{\sum }t_{xy}c_{x,\sigma }^{†}c_{y,\sigma }+\underset{x}{\sum }U_{x}c_{x↑}^{†}c_{x↓}^{†}c_{x↓}c_{x↑}$$

The terms in the *Hubbard Hamiltonian* are:

- x and y are two nearest neighbor sites;
- V is a vertex set that is normally assumed to form a translationally invariant lattice, whose characteristics are important to define the properties of the model [82];
- σ is the spin electron;
- $t_{xy}$ is the transfer or hopping matrix element. It indicates that the dispersion energy band is now expressed in terms of hopping [32];
- c indicates Bloch functions described by the spin index;
- $U_{x}$ is the interaction matrix element of the electron–electron interactions known as Hubbard-U [15,82], which weighs the electron–electron correlation in term of strength when two electrons are placed in the same site [32].

The *Hubbard Hamiltonian* model is composed of two parts: a single-particle component and a two-particle component. The first component is usually called kinetic energy ($H_{kin}$) and defines the hopping of the particles on a lattice [82]. $H_{kin}$ affects the formation of the bands and their delocalization [32]. The second component describes the interaction between two particles and is called a correlation term ($H_{int}$) [32]. The interactions considered in the model are just on-site interactions. In this way, electron correlations due to Coulomb interaction are easily introduced in the model by considering only the onsite Coulomb term (U), the most weighted contribution [28]. The Coulomb interaction is expected to be screened [82]. The description of the hopping and the interactions as single parameters is justified by assuming the transitional invariance of the lattice and allowed hopping only between nearest neighbor sites in the lattice [82]. Extensions of the *Hubbard model* enabling more realistic descriptions are also available [83]. For example, the electron transfers to the next nearest neighbors and the correlation terms between different sites can be incorporated into the *Hubbard model* [32]. An interesting property of the model is that $H_{kin}$ and $H_{int}$ do not favor any type of magnetic ordering per se, while their sum ($H_{kin}+H_{int}$) is believed to produce different kinds of magnetic ordering (including antiferromagnetism and ferromagnetism) and also superconductivity [78]. $H_{kin}$ and $H_{int}$ terms represent two competing mechanisms in which electrons behave as “waves” in the former and as “particles” in the latter [78]. It is this “competition” between the two terms that brings about interesting magnetic phenomena [78]. The model provides rigorous results when it is used to define the magnetic behavior of a material in its ground state [82], and it describes almost all the magnetic phenomena observed in nature, such as magnetic orderings, metal–insulator transition, superconductivity and Fermi liquids [82].

#### 2.3.5. Additional Remarks

It is possible to define a general expression of the Hamiltonian operator $\hat{H}$ of the *Schrödinger equation* (Equation (1)) for *d*-transition metals and their alloys in the absence and presence of an external magnetic field. Equation (24) [33] shows relevant energy contributions that can participate in the stabilization of the ground state energy of materials containing transition metals (see also Equation (2) for a complete mathematical treatment) when no external magnetic field is applied.

(24) $$\hat{H}=T_{e^{-}}^{kinetic}+V_{N+e^{-}}^{Coulomb}+V_{e^{-}+e^{-}}^{Coulomb}+V_{L}+V_{spin-orbit}$$

where

- $T_{e^{-}}^{kinetic}$ is the kinetic energy of the electrons;
- $V_{N+e^{-}}^{Coulomb}$ is the Coulomb attraction between nucleus and electrons;
- $V_{e^{-}+e^{-}}^{Coulomb}$ is the energy factor of the electron–electron Coulomb repulsions;
- $V_{L}$ is the potential due to the crystal field (see references [13,32,33,69] for a detailed description of the crystal field theory in solids); and
- $V_{spin-orbit}$ is the energy factor due to the spin–orbit coupling.

Depending on the metal type and the characteristics of the system, each energy contribution can have a different weight in the expression of the Hamiltonian (e.g., the energy contribution due to the spin–orbit coupling ($V_{spin-orbit}$) might be omitted in the case of *3d* metals, after verifying its negligible participation). Equation (25) [33] includes the energy factor related to the presence of an applied external magnetic field:

(25) $$\hat{H}=T_{e^{-}}^{kinetic}+V_{N+e^{-}}^{Coulomb}+V_{e^{-}+e^{-}}^{Coulomb}+V_{L}+V_{spin-orbit}+\hat{\mu }H$$

where $+\hat{\mu }H$ indicates the energy contribution of the interaction between the electrons of the system and the external magnetic field, which depends on the strength and the direction of the applied magnetic field (H).

### 2.4. Magnetic Properties of 3d-Transition Metals and Their Alloys

Outer *3d*-electrons in pure metals and their alloys are generally considered itinerant (see Section 2.3.2), even though both the localized and itinerant characters can be present simultaneously in the solid, due to the anisotropy of the *3d*-wave functions [27]. The magnetic behavior along the *3d*-transition metals (V, Cr, Mn, Fe, Co, Ni, Cu and their alloys) varies according to the sequential filling of *d*-electron states, a characteristic that is not observed for other transition metals [32,77]. The crystal structure also changes along the series with the *d*-filling [32]. The magnetic and crystal structures of the *3d*-transition metals are:

- Vanadium (V) is a Pauli paramagnetic metal. This indicates that its conduction electrons are weakly magnetic [31]. V possesses a body-centered cubic (bcc) crystallographic structure [14] and becomes a superconductor at 5.265 K [14,84].
- Chromium (Cr) has a body-centered cubic (bcc) structure and it is an antiferromagnetic metal (T_N_~312 K) [14,85].
- Manganese (Mn) is an antiferromagnetic transition metal (T_N_~95 K) and its most stable phase at room temperature is called α-Mn, which has a cubic crystallographic symmetry [14,85]. α-Mn is a peculiar metal for its crystallographic and magnetic characteristics [85].
- Iron (Fe) is a ferromagnetic metal (T_C_~1044 K) with a body-centered cubic (bcc) crystal structure [14,85].
- Cobalt (Co) is a ferromagnetic metal (T_C_~1394 K) and has hexagonal close-packed (hpc) crystallographic structure [14,85].
- Nickel (Ni) is a ferromagnetic metal (T_C_~631 K) having a face-centered cubic (fcc) crystal structure [14,85].
- Copper (Cu) is a face-centered cubic (fcc) solid. It is the only *3d* metal that exhibits a diamagnetic behavior ($\chi /10^{6}\;$= −1.1 at 298 K) [15], since it has a completely filled *3d*-band [72] (the sequential filling of the *d*-shell induces the related bands to become narrower and to energetically shift below the Fermi energy) [32].

**Figure 6:**
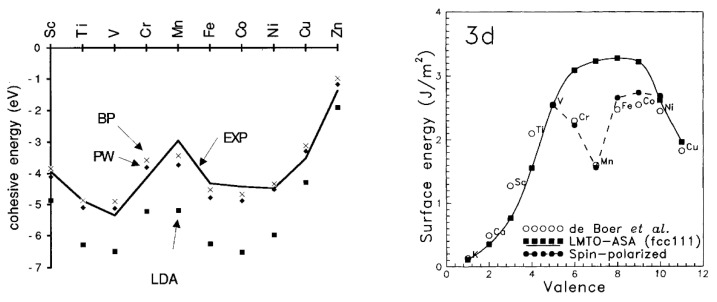
(**Left**): Trend of the experimental (black solid line) and calculated cohesive energies for the *3d* metal period. Solid squares, solid rhombs and crosses indicate the LDA (local density approximation), PW (generalized gradient approximation (GGA), Perdew-Wang, PW91) and BP (GGA, Becke-Perdew) functionals used to calculate the cohesive energy values, respectively. Reprinted figure with permission from P.H.T. Philipsen and E.J. Baerends, Physical Review B, 54, 8, 5326–5333, 1996. Copyright 1996 by the American Physical Society. https://doi.org/10.1103/PhysRevB.54.5326 [90]. (**Right**): Trend of experimental surface energies, derived from surface tensions measurements of liquid metals (open circle), and calculated surface energies for the *3d*-period. Solid squares indicate the surface energies obtained from paramagnetic calculations of fcc (111) surfaces for all the *3d*-periods, while solid circles indicate those obtained from spin-polarized calculations of Cr (bcc (100)), Mn (bcc (100)), Fe (bcc (110)), Co (hcp (001)) and Ni (fcc (111)) surfaces. Reprinted figure with permission from M. Aldén, H.L. Skriver, S. Mirbt and B. Johansson, Physical Review Letters, 69, 15, 2296–2298, 1992. Copyright 1992 by the American Physical Society [91].

The *3d*-transition metals from Cr to Ni are known to express spontaneous magnetism. Some authors pointed out that their magnetic properties are related to a sufficiently smaller radial extension of their *3d*-orbitals [45] compared to the larger ones in V [80]. This, in turn, explains why V possesses paramagnetic behavior without a completely filled *3d*-band. The magnetic properties of *3d* metals are not mirrored in the second and thirds row (*4d*- and *5d* metals), where the transition metals are generally considered paramagnetic, with the exception of silver [14,86,87] (the diamagnetic nature of gold is still under debate) [88]. For example, platinum (Pt) and palladium (Pd) possess the same crystal structure (face-centered cubic (fcc)) and number of valence electrons of Ni, but they do not exhibit spontaneous magnetism [15,77]. This can also be explained by applying the *Stoner criterion* [15]. Moreover, another magnetic interaction becomes relevant in defining their magnetic properties particularly in *5d* metals: this is the *spin–orbit* interaction [15,28,33], which is generally considered negligible in *3d*-electron system [15,28]. The large spin polarization exhibited by *3d* metals is also responsible for their anomalous cohesive energies [89,90] and surface energies [91] in comparison with those of the transition metals of *4d*- and *5d*-series. A two-peak trend can be obtained by plotting the cohesive energies (Figure 6 left) or surface energies (Figure 6 right) vs. the orbital filling for the *3d*-series. A minimum can be seen between two maxima in the middle of the series that usually coincides with half-filled AFM Cr and, more so, Mn [89,91]. A one-peak structure can be instead obtained for *4d*- and *5d*-series with one maximum in the middle of the series [91].

**Figure 7:**
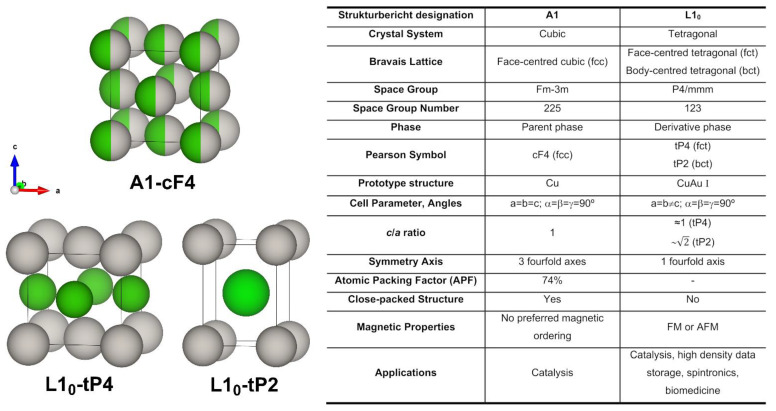
(**Left**): Unit cells of the parent A1 and derivative L1_0_ phases (**left**). Green and gray colors indicate *3d*-elements (e.g., Fe, Co and Ni) and the Pt atoms, respectively. (**Right**): The table shows main crystallographic and general information on the two structures.

Alloys of FM *3d* metals with platinum are known to possess interesting magnetic properties [14,60,86,87,92,93,94,95]. We report intermetallic MPt here, where M can be a ferromagnetic (FM) element such as Fe, Co and Ni, since they are receiving a lot of attention nowadays [5,96,97,98]. Intermetallic MPt can be divided in two main types: disordered (or fcc) and ordered (or fct, face-centered tetragonal). From a crystallographic point of view, tetragonal ordered compounds belong to the L1_0_ phase (Strukturbericht designation) and are made by atomic layers of magnetic *3d* metals and Pt stacked one above the other. The estimated Pt-M plane distances are 1.85 Å, 1.84 Å and 1.82 Å for the bulk structures of fct FePt, CoPt and NiPt, respectively. The face-centered tetragonal phase is a structure that derives from the fcc one and that belongs to the A1 type (Strukturbericht designation). Interestingly, the parent fcc is characterized by a chemically disordered distribution between the *3d* and Pt atoms (i.e., random atomic arrangement), where each crystallographic site can be equally occupied by a *3d* or Pt atom [99]. The unit cells of the bulk structures of A1 and L1_0_ are displayed in Figure 7, along with their main crystallographic features and classifications. A1 and L1_0_ MPt materials can be ulteriorly named using the corresponding prototype structure (Cu and CuAu I, respectively) or Pearson symbols [100] (Figure 7). For example, the Pearson symbol of the A1 unit cell is cF4, where c stands for cubic, F for face centered and 4 indicates the total number of atoms present in the unit cell. The L1_0_ structure can be described in two equivalent ways: tP4 and tP2, where t stands for tetragonal, P stands for primitive unit cell and 4 or 2 indicate the total number of atoms in the unit cells. Further useful crystallography information can be found in the Encyclopedia of Crystallographic Prototypes [101,102,103].

As mentioned before, characteristic examples of L1_0_ systems are fct MPt (M = Fe, Co, Ni) with a M:Pt ratio equal or close to 1:1. L1_0_ phase in M_x_Pt_1−x_ is only formed when M content range is x = ~40–68 for Fe [104,105,106], x = 25–60 for Co [95] and x = 46–50 for Ni [94,107]. Remarkably, the derivative L1_0_ structure emerges as the most stable phase at room temperature (RT) for the 50:50/M:Pt ratio, while the parent A1 becomes stable only at high temperatures [95,99,107,108]. Despite their higher stability, L1_0_ MPt compounds cannot be experimentally prepared at r.t. Thermal treatments are always necessary to transform the chemically disordered A1 phase into the chemically ordered L1_0_ one [95,109,110]. This transformation is a first-order transformation (Ehrenfest designation) [99] and depends on various factors such as the particle shape and size, *3d* metal content (i.e., M:Pt ratio), the temperature and time of the thermal treatment [104,111,112,113]. A useful ally to experimentally monitor the success of fcc-to-fct transformation is the so-called long-range order parameter (S) [114,115]. It provides a quantitative estimation of the degree of the chemical ordering: S = 0 indicates a fully chemically disordered system, while S = 1 indicates a fully chemically ordered one. The S parameter can be calculated by Equation (26) and/or Equation (27) [115].

(26) $$S≅0.85{\left[\frac{I_{001}}{I_{002}}\right]}^{1/2}$$

where (001) and (002) are integrated intensities of the diffraction peaks of the compounds obtained from X-ray diffraction patterns.

(27) $$S=\frac{r_{M}-x_{M}}{y_{Pt}}=\frac{r_{Pt}-x_{Pt}}{y_{M}}$$

where:

- $x_{M}$ and $x_{Pt}$ are the atomic fractions of *M* and *Pt* in the same sample;
- $y_{M}$ and $y_{Pt}$ are the fractions of *M* and *Pt* sites; and
- $r_{M}$ and $r_{Pt}$ are fractions of Fe or *Pt* sites occupied by the correct atomic species.

Another way to estimate the degree of the chemical ordering is by using the ratio between the cell parameters *c* and *a* (i.e., *c*/*a* ratio) of the experimental unit cell. The corresponding values for fcc and fct MPt structures are reported in Figure 7. Table 1 shows some *c*/*a* ratios for intermetallic MPt nanoparticles used as ORR catalyst.

Several experimental [124,125,126,127,128] and computational [129,130,131] studies investigated fcc-to-fct transformation and its factors in MPt systems. L1_0_ MPt alloys, with a composition equal or close to M_50_Pt_50_ and an S parameter close to 1, possess complex magnetic properties. Their most relevant magnetic features are a large magnetocrystalline anisotropy (MCA) constant (K), a high coercivity (H_c_), a ferromagnetic ground state and T_C_ above RT [95,110]. Nevertheless, what makes them appealing for magnetic devices is their ability to retain their magnetic properties in small-sized nanoparticles. Indeed, fct FePt and CoPt can be prepared as NPs as small as ~3 nm and still be chemically stable ferromagnets [109,112,132]. To be appealing in practical applications, these compounds should satisfy the requirement that $K_{u}V≅60k_{B}T$, where $K_{u}V$ is the stored magnetic energy and $k_{B}T$ is the thermal energy ($K_{u}$ is the uniaxial magnetocrystalline anisotropy constant, V is the volume of the magnetic domain, $k_{B}$ is the Boltzmann constant and T is the temperature in Kelvin) [112,133].

At the root of their peculiar magnetic properties, there is a strong interplay between the spin magnetic moment, the orbital magnetic moment and the total magnetic moment (spin + orbital moment) of M and Pt with the crystal lattice through the orbital component [15,110,134]. Magnetocrystalline anisotropy mainly arises at the microscopic scale from spin–orbit interactions [15,134]. MCA is larger in crystal systems with low symmetry such as L1_0_ [110,126,127,135] and smaller in high-symmetry ones, such as A1 [15,110,134]. For example, the highest MCA energy in L1_0_ FePt is reached at the peak of its tetragonality (i.e., *c*/*a* ratio fully satisfies the requirement of tetragonality, thus when S = 1; see Figure 7) [110,136]. MCA and spin–orbit coupling have been extensively investigated in MPt systems both experimentally [105,126,127,135] and computationally [137,138,139,140,141].

Physical and magnetic properties of A1 and L1_0_ bulk structures are listed in Table 2 for MPt systems (M = Fe, Co, Ni); these properties include disorder–order critical temperature, magnetic ground state, T_C_, uniaxial MCA constant (K_u_) (FM L1_0_ have one easy axis of magnetization along c-axis, [0 0 1] direction) [99] and saturation magnetization (M_s_). Table 2 also shows that all MPt alloys have an FM ground state, with the exception of L1_0_ NiPt. A1 phases are classified as soft magnets (i.e., easy magnetization and demagnetization), while L1_0_ structures belong to the family of hard magnetic materials (i.e., hard magnetization and demagnetization) [15]. This is indeed the reason why L1_0_ FePt and CoPt are investigated for technological applications, since their hard magnetic properties make them exploitable as permanent magnets [15,142]. A relevant and experimentally accessible figure-of-merit to measure the degree of magnetic hardness is the maximum energy product, (BH)_max_ [142]. (BH)_max_, together with S parameter, may be a useful quantity in the preparation of L1_0_ MPt systems.

Regarding the interesting case of L1_0_ NiPt, some authors explained that the absence of a magnetic ordering is due to the susceptibility of Ni magnetic moment to its chemical environment (i.e., number of nearest neighbors, NN) [94]. In other words, A1 structure possesses up to 12 possible magnetic NNs per Ni atom, while the NN number is reduced to only four in L1_0_, despite both structures exhibiting the same Ni:Pt ratio [94]. As a consequence, more than four magnetic NNs per Ni atom are needed to exhibit ferromagnetism in NiPt alloys [94]. This large local environmental effect in Ni-Pt alloys has also been confirmed by computational studies [143].

**Table 2:** Magnetic and structural properties of MPt (M = Fe, Co and Ni) bulk materials. O and D stand for chemically ordered and disordered phases, respectively. Magnetic ground states are indicated as FM (ferromagnetic) and P (paramagnetic). Magnetic quantities are reported in the centimeter/gram/second system (CGS).

	FePt		CoPt		NiPt	
**Strukturbericht Designation**	A1	L1_0_	A1	L1_0_	A1	L1_0_
**Chemical Ordering**	D	O	D	O	D	O
**Order–Disorder Critical Temperature** **(K)**	~1573 [99,108]		~1106 [95]; ~1098 [99,144]		~940 [128,144]	
**Heat of Formation (** **ΔH_f_) (eV/atom)**	-	−0.73 ^a^ [145]	-	−0.140 [146]	-	−0.096 [128]
**Magnetic Ordering**	FM [108]	FM [108]	FM [147]	FM [147,148]	FM [94]	P [14,94]
**Curie Temperature (T_C_)** **(K)**	585 [108]	750 [108]; 670 [14]	-	750 [14]; 710 [149]; 850 [133]	-	-
**Magnetically**	Soft	Hard	Soft	Hard	-	-
**Maximum Energy Product (BH)_max_ (MGOe)**	-	~13 [150]	-	~9.7 [95]	-	-
**Uniaxial MCA Energy Constant (K_u_)** **(10^7^ erg/cm^3^)**	$<K_{u}^{L1_{0}}$ [110]	7 [119]; 6.6–10 [133]	<4.9 [126]	4.9 [133]	-	-
**Saturation Magnetization (M_S_) (emu/cm^3^)**	-	1140 [133]; 1150 [151]	-	800 [133]	-	-
**Minimal Stable Grain Size (D_p_) (nm) ^b,c^**	-	3.3–2.8 [133]	-	3.6 [133]	-	-

^a^ Obtained with CALPHAD (CALculation of PHAse Diagrams) method; ^b^ Dp=(60kBT/Ku)13 where T = 300 K, k_B_T = 3.77·10^−14^ erg; ^c^ calculated stability of “cubic” grain sizes of the material over 10 years for density magnetic recording application based on the media stability criterion (K_u_·V ≥ 60·K_B_T).

## 3. Catalysis and Magnetism

**Figure 8:**
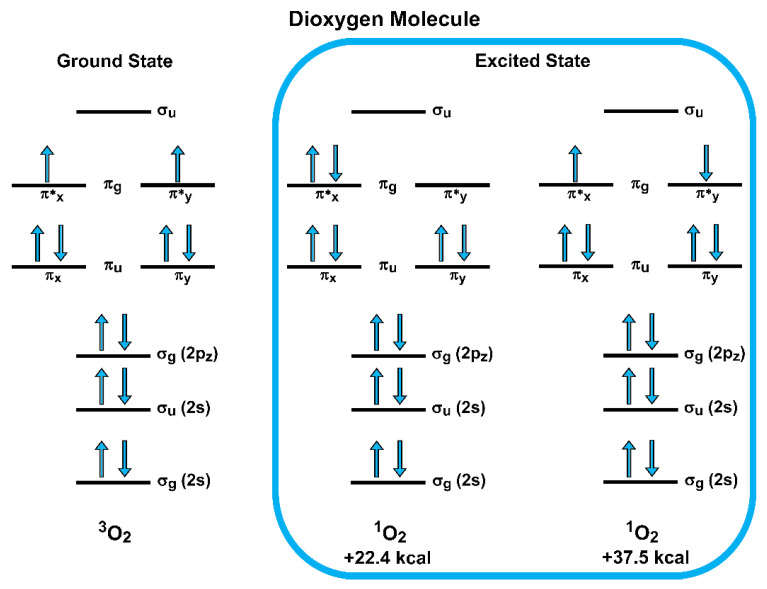
Electronic configurations energy levels of the ground state ^3^O_2_ (triplet oxygen) and excited state ^1^O_2_ (singlet oxygen) of O_2_ molecule.

One of the most important molecules for life and for industrial processes is dioxygen (O_2_). Its ground state is triplet oxygen (^3^O_2_), characterized by two unpaired electrons aligned in a parallel manner (Figure 8, left). Triplet oxygen is a paramagnetic molecule and, despite being a diradical, is less reactive compared to its diamagnetic and less stable singlet state (^1^O_2_) [152,153,154] (Figure 8, right). The features that make ^3^O_2_ unique are an unusual presence of strong π bonds and weak σ bonds [155] and two aligned unpaired electrons [156]. When ^3^O_2_ reacts with a catalytic system, such as enzymes or solid catalysts, two processes are needed to convert ^3^O_2_ rapidly into the products: weakening O-O bond and interconverting the spin of the unpaired electrons. The strong π O-O bond can be weakened by transforming the triplet into the singlet species (for example, by using light) or by reducing it in a sequence of one-electron reductions [156,157]. A different and more complicated task is, instead, the spin inversion (spin flip), since it is a slow process and a “forbidden” reaction [158]. When ^3^O_2_ reacts with common diamagnetic systems (closed-shell compositions), this spin inversion must occur during the chemical transformation [157,159]. On the contrary, when ^3^O_2_ reacts with materials with unpaired electrons (i.e., open-shell compositions, magnetic systems), this step is not necessary, since the spin restriction is removed or becomes negligible in this case [157]. However, in the latter case, the O-O bond is conserved intact in the first reaction steps and broken in the following ones [157]. ^3^O_2_ and its reactivity are a simple example of the key role played by the spin/magnetism in catalysis [160,161,162,163,164].

### 3.1. Oxygen Reduction Reaction (ORR)

The understanding of the ORR mechanism, one of the major causes of the activation overpotential in fuel cells, has been the subject of extensive investigations both experimentally [2,165,166,167,168,169,170,171] and computationally [172,173,174,175,176,177]. The various complex multi-steps and parallel reactions required to reduce the oxygen gas depend mostly on the pH of the electrolyte (acidic or alkaline media) and on the chemical and structural composition of the catalyst (e.g., the presence of structural defects on the catalysts surface such as vacancies, step atoms and terraces) [169,178]. Other factors must be considered as well: for example, the formation of some intermediate species considered as the rate determining steps of the process (e.g., some adsorbed oxygen and hydroxyl species) [170,179], the coverage of adsorbed oxygen atoms onto the surface catalyst [169], the electrolyte solution [176] and the operating parameters such as temperature and pressure [2,171,173].

The desirable ORR pathways are those that lead straight to the formation of water molecules, since it is an environmentally friendly product and it does not cause damage to the fuel cell. Undesirable parallel and competitive pathways are the ones activating the O-O bond in a fast fashion and causing a reduction in the catalyst lifetime by producing corrosive H_2_O_2_ as an intermediate [172]. Generally, two different simplified ORR mechanisms are described in the literature: the direct four-electron (4e^−^) reduction, which leads to the direct formation of water molecules (^3^O_2_ + 4H^+^ + 4e^−^ → 2H_2_O), and the indirect two-electron (2e^−^) reduction, which produces first the undesired hydrogen peroxide (^3^O_2_ + 2H^+^ + 2e^−^ → H_2_O_2_) and then later the desired H_2_O molecules (H_2_O_2_ + 2H^+^ +2e^−^ → 2H_2_O or 2H_2_O_2_ → 2H_2_O + ^3^O_2_).

**Figure 9:**
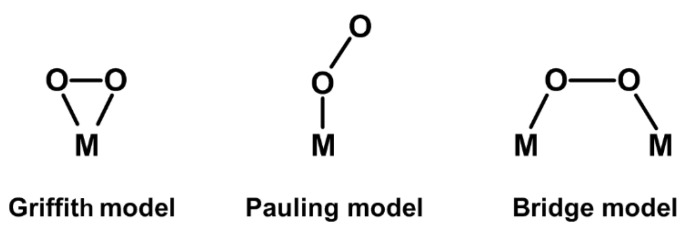
Suggested models for the adsorption of the oxygen molecule onto a metal catalyst (M) surface.

More realistic and complete pathways have been proposed over the years; they involve the formation of various intermediates such as H*, O*, O_2_*, OH* and OOH*. The first ORR step corresponds to the adsorption of the oxygen molecule (^3^O_2_) onto the catalyst surface. According to the literature, three possible models [180] (Figure 9) have been suggested:

- The Griffith model, in which both oxygen atoms interact with a single atom of the catalytic surface (the less common type of adsorption);
- The Pauling model (or end-on configuration), in which only one of the two oxygen atoms is coordinated with one atom of the catalytic surface; and
- The bridge model, in which two bonds are formed involving both O atoms with two different atoms of the surface.

**Figure 10:**
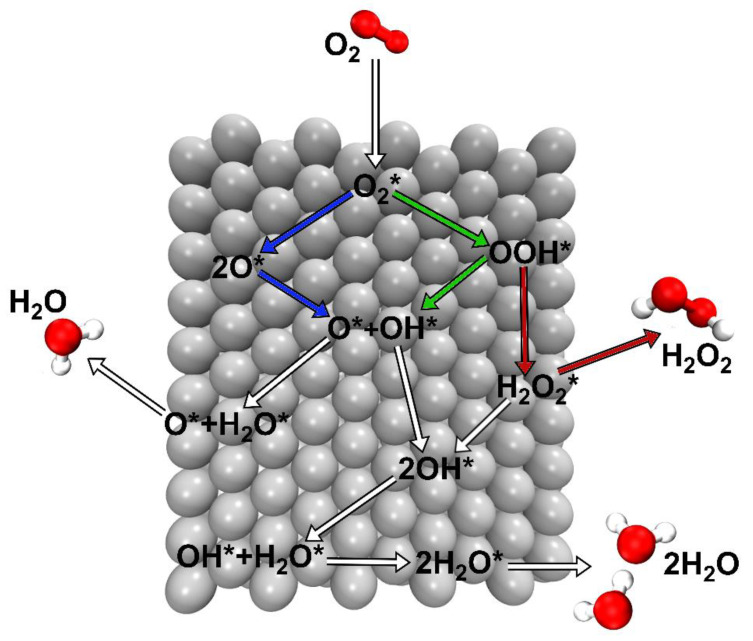
Sketch of proposed ORR mechanisms onto platinum as catalyst and relative products. The so-called associative and dissociative mechanisms are indicated by green and blue arrows, respectively. The formation of undesired hydrogen peroxide H_2_O_2_ is shown with red arrows. White arrows indicate the common steps.

The bonding pattern of the oxygen molecule onto the catalytic surface depends on the surface geometry of the catalyst and on the binding energies [176,181,182]. Adsorption of ^3^O_2(g)_ always occurs without cleavage of the O-O bond, forming the O_2_* species. ORR can proceed after this step via two different yet interconnected pathways. The first is the so-called dissociative pathway, characterized by the cleavage of the O-O bond of O_2_* species into adsorbed oxygen atoms (O*), that, in turn, experience various reaction steps resulting in the production of water molecules. The second pathway is known as the associative pathway: O_2_* species undergoes a proton transfer, forming OOH* species. OOH* can then follow different transformations including the generation of the corrosive and undesirable H_2_O_2_ molecule. Figure 10 shows some of these possible ORR pathways in the case of catalytic platinum in a proton exchange membrane fuel cell [173].

Other plausible pathways can also exist—some authors state that O_2_* can achieve a “superoxo” state ($O_{2}^{-}$) or a “peroxo” state ($O_{2}^{2-}$) before the cleavage of the O-O bond [173]. Experimental evidence of the formation of superoxide anion ($O_{2}^{-}$) has been found on the surface of Pt electrodes in both alkaline and acidic electrolytes-based fuel cells, as well as the possibility that it plays an important role in the first ORR step [183].

ORR cannot take place without the presence of hydrogen atoms, which can react with oxygen (O* and O_2_*) and intermediate (OH* and OOH*) species. Two different approaches are usually considered to computationally investigate how these reactions occur at the surface: the Langmuir–Hinschelwood (LH) mechanism and the Eley–Rideal (ER) mechanism [173]. According to the LH mechanism, the molecules of the reagents are adsorbed on neighboring sites of the surface and then react in a bimolecular reaction. According to the ER mechanism, only one of the reactants is chemisorbed on the surface, while the other one reacts directly from the gas phase without being adsorbed. In the case of fuel cells, the LH mechanism expects that hydrogen atoms react directly after adsorption on the surface, while the ER mechanism assumes that hydrogen atoms interact with O* through the electrolyte solution [173]. The LH mechanism is generally the most used approach to investigate the ORR mechanism in fuel cells.

Although ORR is not a complex reaction, the detailed comprehension of the various steps of this metal-catalyzed reduction still represents one of the most important topics to design and develop targeted catalysts at the cathode side in fuel cells.

### 3.2. Applications of 3d Metals and 3d-Based Alloys

**Figure 11:**
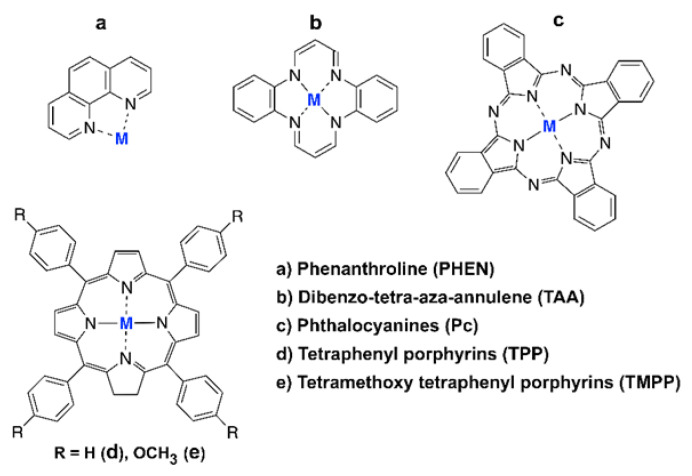
Molecular structures of some the most investigated macrocycles as NPM catalysts. M stands for Mn, Fe, Ni, Co or Cu.

The first catalyst employed in fuel cells was platinum, and it is still the most used metal nowadays, due to its favorable catalytic properties, especially in improving the efficiency of the sluggish ORR [2,3,184]. However, platinum is a scarce, noble and expensive metal. Two possibilities are feasible at this point: the decrement in its rate of usage (i.e., Pt loading) and its partial/complete replacement with other noble, but less expensive metals [12] (e.g., Pd and its alloys [185] or Au [186]) or with abundant and non-precious metals (NPM) (e.g., *3d*-transition metals) [12,20,98]. The first solution has been already applied by downscaling the Pt loading from 28 mg/cm^2^ (previous fuel cells models) to 0.3 mg/cm^2^ [187], with room for further possible downscaling (e.g., the U.S. DoE 2020 goal was to reach a Pt group metal loading of 0.125 mg/cm^2^) [2] by using materials that combine Pt with other metals (e.g., alloys and core–shell structures) or by changing the design of the material (e.g., Pt-monolayered materials, one-dimensional nanowires and nanotubes) [12,20,165,184,188,189,190]. The second solution is extensively under investigation and involves the use of different NPM-based catalysts [12,20,184,188,191]. Some example are macrocycles (few relevant mentions in Figure 11), electroconductive polymers (e.g., *3d*-transition metals conjugated with heterocyclic polymers such as polyaniline and polypyrrole) [192], transition metal-based inorganic nanoparticles (e.g., metal carbides, metal nitrides, metal oxides and metal chalcogenides) [12,165,184,188] and metal-free catalysts (e.g., carbon-based compositions doped with heteroatoms such as N, B, P, S) [12,165,184,188].

In any case, curbing costs is not the only desirable feature of a potential Pt substitute. Without a doubt, an optimal catalyst for fuel cells must ensure suitable ORR activity (equal to or greater than pure platinum), concomitantly showing adequate stability at the operating conditions [193]. High performances, long durability and low costs are the three major necessary perks for a large-scale commercialization of PEMFCs, most of all in the automotive sector [5,6]. The search for a replacement for Pt that improves these three perks represents one of the main challenges in the development of this technology.

### 3.3. Bimetallic Pt-Based Alloys as ORR Catalysts

Currently, the most successful strategy to identify a suitable fuel cell catalysts with suitable ORR activity and stability relies on combining the catalytic properties of Pt with a second (or more) different heterometal, such as *d*-block transition metals (e.g., Fe, Co, Ni, Cu, Sc, Y, Zr, Hf, Pd) [12,98,193], post-transition metals (e.g., Pb) [12], lanthanides (La, Ce, Pr, Sm, Gd, Tb, Dy, Tm) [194] and alkaline earth metals (e.g., Ca, Sr) [195].

Among all the possible combinations, Pt-bimetallic alloys such as Pt-Co [12,196], Pt-Ni [12,196], Pt-Cu [12,197] and Pt-Y [198,199,200] are the most widely studied, both experimentally [12,98,193,196,201,202,203] and computationally [199,204,205,206,207], since they exhibit sufficient catalytic activity in ORR. The most interesting feature of Pt-bimetallic compositions is that the alloy displays different properties compared to the pure homometallic parents. Likewise, different properties are usually more enhanced when the heterometal is placed in the sublayers of a Pt-enriched surface (Pt-skin surface) [193,208,209,210]. The presence of heterometal atoms below the Pt-skin induces several modifications to the surface atoms, and thus to the catalytic behavior of the solid catalyst. Such variations are the result of chemical, electronic and physical perturbations (generally investigated through computational chemistry) and are collectively named chemical effects in heterogeneous catalysis [178]. Chemical effects comprise the ligand [209,211], the strain [212] and the ensemble [178,213] effects. Depending on the type, the stoichiometry and the distribution of the heterometal centers in the catalyst structure, these chemical effects are assumed to influence its electronic properties by tuning the chemisorption properties at the surface [204,205,209,211]. The ligand effect is referred to as the changes in the electronic environment induced by the hetero metal centers through the metal–metal bonds; more in general, the ligand effect describes modifications of the chemical properties of the surface atoms due to alloying [213]. The strain effect is related to fluctuations in the metal–metal bond lengths between various layers in the solid structure and imposes strained overlayers characterized by a mismatch of the lattice constants within the catalytic layer. Thus, the ligand effect changes the catalytic activity through perturbation in the electronic interactions between the components of the bimetallic alloy [208,214], while the strain effect participates by modifying the orbital overlap [205]. The ensemble effect, or geometric effect, indicates modifications in the geometry of the adsorption site at the surface, due to the presence of the heteroatoms in the sublayers; these modifications may consequently change the activity of the catalyst. The influence of these three phenomena on the catalytic activity of bimetallic alloys is commonly studied computationally, since the experimental characterization of the whole layered structure in such compositions is still limited [215].

Unfortunately, Pt-based alloys are known to exhibit dissolution and degradation problems in the operating environment of fuel cells (PEMFCs), with a concomitant loss of catalytic activity and performances [193,210,216]. For instance, the heteroatoms may diffuse to the surface (segregation phenomenon [217]) or the Pt atoms at the surface may dissolve in the electrolyte, thus exposing the sublayer to direct contact with the reagents [193]. Furthermore, some computational studies reported that the structural stability of a catalyst may also be affected by the presence of adsorbed species (for example, O* or OH*) that could facilitate the exchange [218,219] and/or the removal of Pt atoms from the surface [210,220]. This creates vacancies where the sublayer heteroatoms become directly exposed to potential adsorbed species [220], delivering the formation of strong bonds (i.e., an adverse catalytic step in ORR mechanism) [221]. The influence of the adsorbed species on the stability of the catalyst depends, in turn, on the adsorbate coverage. Some authors reported the dissolution of Pt atoms into the electrolyte when the O-coverage is greater than 0.5 ML (monolayer) in bimetallic Pt-based alloys [220], as well as a thermodynamically favored segregation of the hetero-components [210,218]. This said, since stability depends on all these factors, it is a criterion that must be evaluated case by case from both an experimental [193,216,222,223] and computational [224,225,226] perspective.

**Figure 12:**
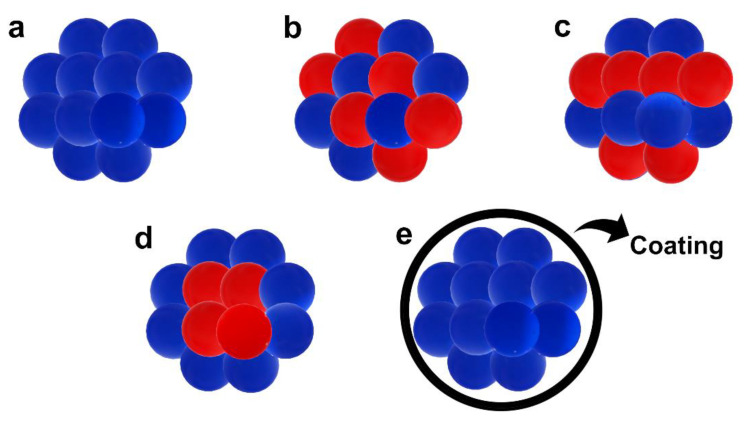
Common Pt-based NPs: (**a**) a simple metallic NP, (**b**) a random bimetallic NP, (**c**) an ordered bimetallic NP, (**d**) a core–shell NP and (**e**) a coated or encapsulated metallic NP.

The most studied Pt-based bimetallic alloys both experimentally [7,111,227,228,229,230,231,232,233,234] and computationally [204,205,206,235,236,237,238] are those containing *3d*-transition metals as heteroatoms. Different Pt_x_M_y_ compositions and various surface facets have been tested as potential substitutes for pure Pt, but generally, Pt_3_M, PtM and PtM_3_, in which M is a *3d*-transition metal (M = V, Cr, Mn, Fe, Co and Ni) nanocatalyst (alloys, intermetallic, nanoparticles and core–shell nanostructures), have proven to be potential suitable candidates, thanks to their adequate ORR activity and stability [96,97,201,202,228,234,239,240,241]. Figure 12 shows common morphologies seen in Pt-based nanoparticles (NPs).

Pt-Fe, Pt-Co and Pt-Ni compositions are considered the best Pt-based catalysts of all the *3d* metal Pt-M series [227,228,229,242,243,244,245]. Indeed, intermetallic Pt-M (M = Fe, Co and Ni) compositions are exploited as catalytic materials in commercially available FCEV for their enhanced catalytic activity and stability over time [5]. Available experimental data on the ORR activity of intermetallic MPt (M = Fe, Co and Ni) ordered (fct) and disordered (fcc) NPs are reported in Table 3, in comparison with commercially available Pt catalysts.

Other compositions such as Pt_3_V(111) and Pt_3_Cr(111) are also claimed as potential catalysts for their solidity and durability under the fuel cell operating conditions [202,252,253]. Pt_3_Mn(111) is the least investigated as a catalyst, even though it has also been studied [254]. The (111) facet in Pt_3_M compositions is generally preferred for experimental and computational research due to its high activity toward ORR compared to other surface facets of the same composition, such as (100) or (110) [176,180]. Moreover, some authors claim that such orientation is the most conductive in small nanoparticles in the case of ORR [255]. Interestingly, all the *3d* metal Pt-based alloys possess a Pt-skin surface as a common structural feature: Pt-skin organization has been confirmed by experimental and theoretical studies to be the most thermodynamically stable and active possible arrangement [193,202,206,228,240,256,257]. Despite their favorable ORR activity, Pt-based alloys, however, can still exhibit stability issues [7,193,219,222].

Why do *3d* metal Pt_-_M compositions and, more in general, *3d* metal-based catalysts promise to be better catalysts for fuel cells than several other investigated compositions? The answer may be a trivial one: *3d*-transition metals are magnetic centers, and the catalytic properties of *3d metal* compositions can be fully understood only by including the complex phenomenon of cooperative magnetism into the investigation.

### 3.4. Catalytic Trends and Magnetism

**Figure 13:**
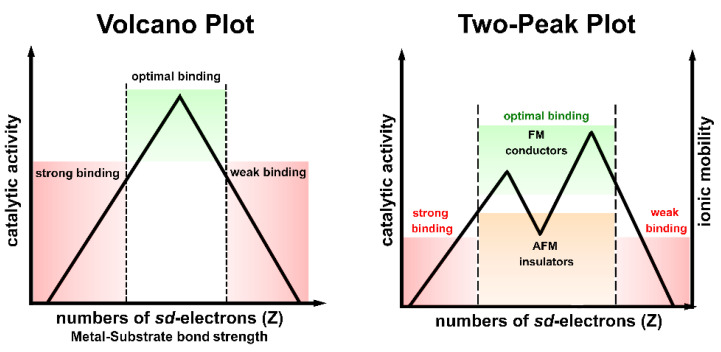
Schematic example of a volcano plot (**left**) and of a multipeak plot (**right**). The volcano-shaped curve shown refers to a general catalytic process, obtained by plotting the catalytic activity vs. the respective metal–substrate bond strength for a series of metal catalysts. Instead, the two-peak structure describes the catalytic activity trend of *3d* metal-based catalysts with comparable coordination and oxidation states vs. the oscillation of their magnetic properties correlated to the orbital filling [19]. Adapted with permission from Biz, C.; Fianchini, M.; Gracia, J. Strongly Correlated Electrons in Catalysis: Focus on Quantum Exchange. *ACS Catalysis* 2021, 11 (22), 14249–14261. Copyright 2021, American Chemical Society.

The suspect that a relationship exists between magnetic properties of the catalyst and catalytic activity in heterogeneous catalysis has a long history [221,258,259,260]. The topic has been known since the early 20th century and it has raised the interest of the scientific community. In his work called “*Magnetism and catalysis*”, published in 1946, P. W. Selwood wrote, “It cannot be denied that those chemical elements which show the most pronounced catalytic activity, namely, the transition group elements, are also the elements which show the most interesting magnetic properties” [258]. The same concept was reaffirmed in 1978 by J. T. Richardson: “Of the “magnetic” catalysts—those susceptible to magnetic measurement—almost all belong to the first, second and third transition series. It is perhaps no small coincidence that the property making these materials paramagnetic—the presence of unpaired electrons—also enables them to form chemisorption bonds and exchange electrons in redox reductions” [259]. Volcano plots were introduced by Balandin [221] as graphic translations of *Multiplet Theory* to try and find a relationship between the electronic properties (which also include magnetic features) of a certain catalyst and its catalytic activity. He pointed out the existence of a “structural and energetic correspondence between the reacting molecule and the atoms of the catalyst” [221]. A volcano plot for a specific catalytic process is built by considering energy factors that are independent of the catalytic nature (such as number of *sd*-electrons (Z)) and factors that are dependent on it (such as catalytic activity). The best catalytic system in a pool of potential candidates ranks at the maximum (or close to it) of the volcano peak, where the structural and energetic factors both match the high catalytic performance. Figure 13 (right) shows an example of a volcano-shaped curve related to a generic catalytic process obtained by plotting catalytic activity vs. the number of *sd*-electrons (Z) (or metal–substrate strength). The curve indicates that for the chemical transformation under study, the catalysts that bind the substrate too weakly or too strongly on the surface possess a low catalytic activity (“bad” catalysts), while the catalysts with milder binding (neither too weak nor too strong) exhibit higher catalytic activity (“good” catalysts). This interpretation also satisfies the Sabatier principle [178,261], which states that a process occurs when the interactions are “exactly right”. For this reason, volcano plots are useful tools in heterogeneous catalysis and are widely applied to select the best catalyst in terms of activity and selectivity for catalytic processes (e.g., ORR) within a pool of potential candidates (generally among closed-shell nonmagnetic catalysts) [19]. Despite this, Balandin himself pointed out in various examples [221] that magnetic *3d* metal catalysts, as well as *4f*-electron systems, do not follow the same volcano-shaped curve (Figure 13, left). Some authors proposed instead a two-peak plot (Figure 13, right) to explain such a different trend, especially in the case of *3d* metal-based catalysts [19]. However, the basic concept of a volcano-shaped curve, as defined by Balandin, does not change—the most active magnetic catalyst for a given catalytic process is found at the top of the two peaks.

Figure 13 (right) shows that the best catalysts possess dominant ferromagnetic interactions. Moreover, this two-peak plot is not new for magnetic *3d* metals since it can be also found in the case of cohesive and surface energy trends for these metals, as described in Section 2.4 (Figure 13). In recent years, magnetic compositions have gained a lot of attention and found applications in several industrially relevant chemical processes [241,262,263,264,265] and in other fields [266], such as spintronics devices [267], medicine [268] and gas sensing [269].

Nowadays, there are three possible ways to exploit magnetism in catalysis and in modern technological devices (such as fuel cells):

1. By engineering magnetic catalysts through the increment in their “internal” magnetic properties ($\vec{H_{0}}$) (*intrinsic fields*);
2. By the application of an external magnetic field ($\vec{H}$) (*extrinsic fields*); and
3. By combining the previous two options ($\vec{H_{0}}+\vec{H}$).

**Figure 14:**
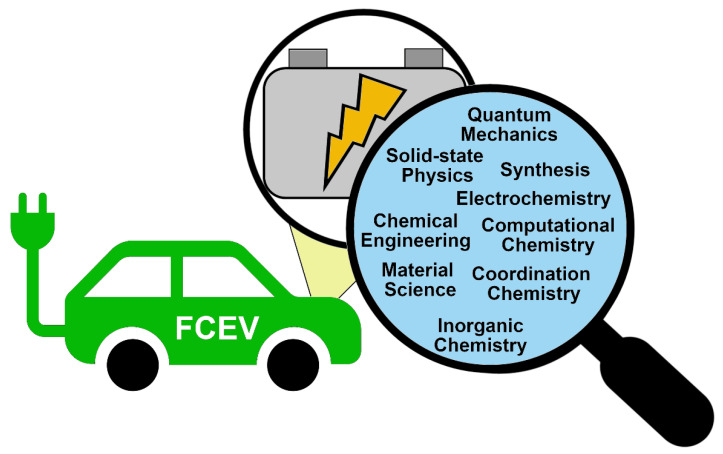
Disciplines involved in the development of PEMFC in fuel cell electric vehicles (FCEVs).

It should be mentioned that interdisciplinary knowledge is required to fully comprehend and exploit the relationship between catalytic structure and electronic properties, intrinsic magnetic phenomena, external magnetic field and catalysis (homogeneous and heterogeneous). Quantum mechanics, physics, solid-state physics, computational and experimental chemistry, electrochemistry and chemical engineering are just some of the disciplines involved in this quest to fully exploit fuel cells (particularly PEMFC) in fuel cell electric vehicles (FCEV), as summarized in Figure 14.

### 3.5. Improvement of Magnetic Properties of Catalysts ($\vec{H_{0}}$, Intrinsic Magnetism)

The study of the magnetic properties of metal ions is known as *magnetochemistry* [270,271]. When these metal ions are involved in catalytic events, their investigation becomes more complex [258]. Thus, the improvement of the catalytic performance of a magnetic catalyst by enhancing its intrinsic magnetic properties is not a simple task. Several synergistic tools can help facing this challenge, such as rational design of the catalysts structure, theoretical/computational studies and experiments. Rational design represents a powerful and useful approach to start solving this task. It requires a deep knowledge of the structure–performance and the electronic structure–catalytic activity relationships [20,132,272,273]. Most of the widely used and investigated catalysts to date have been identified through a trial-and-error strategy. This is not an efficient strategy, by any means, since there may not be enough time and resources to investigate the extensive pool of viable candidate materials. A more cost-effective systematic approach involves the application of modern quantum mechanical methods as screening tools, followed by experimental scoring of the theoretical results and models [274,275,276]. First principle modeling of materials (open- and closed-shell compositions) at the atomic scale represents a powerful tool to search for potential catalysts with an improved catalytic activity and selectivity [178]. Many consolidated computational approaches are available in the scientific literature nowadays [43,277,278,279,280].

**Figure 15:**
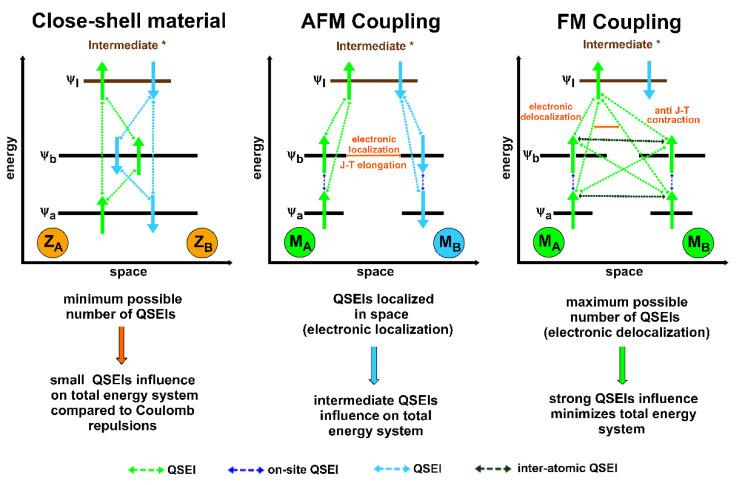
Simplified energy/space plots showing quantum spin exchange interactions (QSEIs) of catalysts and intermediates with a closed-shell ground state (left) and with predominant AFM (center) or FM (right) couplings. The model is strictly limited to a spin orbital $\psi_{I}$, mostly localized in an intermediate of the catalytic process, and to the highest energy levels of the catalysts, namely $\psi_{a}$ and $\psi_{b}$. QSEIs are displayed with colored dotted arrows [19]. Adapted with permission from Biz, C.; Fianchini, M.; Gracia, J. Strongly Correlated Electrons in Catalysis: Focus on Quantum Exchange. *ACS Catalysis* 2021, 11 (22), 14249–14261. Copyright 2021, American Chemical Society.

The choice of the procedure depends on the material, on the chemical and/or physical properties to investigate, on the quality/quantity of information to be collected and on availability/power of computational resources. Another important tool is pure theoretical investigation and speculation (not necessarily carried out and/or supported by calculations), since magnetism can only be understood through quantum mechanics. Towards this goal, some authors introduced the concept of quantum spin exchange interactions (QSEIs) to explain how (indirect) exchange interactions, responsible for creating magnetic ordering in collective magnetism, influence the chemisorption properties of magnetic catalysts and how FM compositions (followed by A-type AFM materials) are the most active ones in some catalytic transformations [19,56,206,281,282,283]. In this regard, Figure 15 shows a simplified sketch of QSEIs in closed-shell, dominant antiferromagnetic and ferromagnetic (open-shell) catalysts. The concepts proposed by J. Gracia and co-authors are nowadays considered part of modern strategies to design optimum magnetic catalysts for ORR [284,285] and OER [286,287,288]. It is worth mentioning that exchange interactions are also claimed to play a role in the so called *spin catalysis* [164,289] and are included in the concept of *exchange-enhanced reactivity* [290,291] in homogeneous catalysis and biochemistry.

The concepts proposed by J. Gracia reflect what was already known through experiments [258,259,272,292]. For example, G. Cohn and J. A. Hedvall experimentally observed a decrement in the catalytic activity of a Co-Pd alloy in the decomposition of formic acid, when the catalyst experienced the transition from ferromagnetism to paramagnetism in the absence of an applied external magnetic field [293]. They pointed out that the ferromagnetic state in the catalyst for the decomposition of formic acid allows a decrement in the activation energy of the reaction of about 30% with respect to its paramagnetic state [293]. This change in the catalytic activity due to the change in the magnetic properties of the catalyst (i.e., the catalyst undergoes a magnetic transition) is known as the *internal magneto-catalytic effect,* or *Hedvall effect* [294], and it is connected to the destruction of the exchange forces among neighboring atoms in the catalyst lattice [293,294,295] (see Figure 15 right on QSEIs), which are responsible for the ferromagnetic ordering. Other examples of this *internal magneto-catalytic effect* were also reported in the literature [295,296,297,298]. It is also worth mentioning that conflicting results were observed in the Ni carbonylation to form Ni(CO)_4_ complex (diamagnetic) when Ni reacts with gaseous CO (Ni + 4CO → Ni(CO)_4_) at atmospheric pressure. In this case, in fact, a Ni_1−x_Cu_x_ alloy served as the catalyst, in which the Cu concentration (x) was varied in order to fit the change in the magnetic phase (T_C_) with the range temperature of the reaction (298 K < T < 443 K). The authors reported that for the Ni_0_._72_Cu_0_._28_ sample, whose T_C_ falls exactly in the temperature range of the reaction, a decrement in the activation energy is noticed from 0.35 ± 0.05 eV to 0.15 ± 0.05 eV when the sample goes from ferromagnetic to paramagnetic [299]. A.B. Cardwell [300] and D.A. Dowden [272] pointed out that this decrement in the activation barrier may be only indirectly linked to the magnetic transition, and more so due to entropic factors in the activation of both spin channels (i.e., majority and minority of spins).

Such experimental evidence on the *internal magneto-catalytic effect* (or *Hedval effect*) suggests that the comprehension of the correlation between the bulk magnetization and the reactivity of the catalytic process could play a relevant role in modeling electronic interactions for catalytic reactions where the catalysts exhibit magnetic ordering [293,296,299]. This has been extensively remarked by Gracia and co-workers in previous works [19,206,236]. These previous examples show the importance of the synergy between theory and experiments. The synthesis of magnetic catalysts is indeed a complex task, since the methods of preparation influence the composition, the structure, the size and the shape of the magnetic catalyst [132,189,231,259,266,301,302,303]. For example, Pt_3_Co NPs supported on carbon exhibit an increase in the specific activity toward ORR (at 0.9 V vs. RHE, T = 333 K and rotation speed = 1600 rpm) and a parallel decrement in the mass activity, passing from the small size of 3 nm to 9 nm [231]. A compromise between specific and mass activity was found for Pt_3_Co NPs with a size of 4.5 nm [231]. Another study on Pt_3_Co/C shows that NPs, prepared via the solid phase method but annealed at two different temperatures (773 K and 973 K in a H_2_/Ar atmosphere) with comparable size (4.4 nm and 5.1 nm for the sample annealed at 773 K and 973 K, respectively), show different ORR catalytic performances [234]. In particular, the sample annealed at 973 K exhibits a higher activity and a better stability than the other sample [234]. Pt_1−x_M_x_ alloys are known to undergo a structural transformation when they undergo a thermal treatment, from a disordered (fcc phase) to a more ordered phase [95,109,110]. This process depends on several factors other than the annealing temperature [104,111,112,113]. Several other works show that the ordered phase of these Pt_1−x_M_x_ alloys (M = Fe or Co) possesses an improved catalytic activity toward ORR and an improved stability under catalytic operating parameters of the fuel cell [117,229,232,233,240,242,243,244,246,250,304,305,306,307]. The catalytic activity and stability of these NP catalysts can be modulated simply by changing the chemical ordering during the synthesis, while maintaining the same size and composition of the NPs. Despite this, a complete picture cannot not be obtained if magnetic properties are not fully considered, as expressed by the fundamental electronic structure–catalytic activity relationship in catalysis [272]. For example, superparamagnetic fcc FePt/C and ferromagnetic fct FePt/C NPs of comparable size exhibit different ORR catalytic activity, being the FM catalysts the most active [308]. Indeed, compositions such as ordered fct FePt and fct CoPt, as described in Section 2.4, possess peculiar magnetic properties, including high (BH)_max_ and strong MCA; these magnetic properties depend on the preparation method, composition, size and shape of the nanoparticles [132,309]. Table 4 shows available experimental data on MPt (M = Fe, Co) nanoparticles and how their magnetic properties differ with the preparation method, the stoichiometry and structural parameters: the magnetic proprieties considered in the table are the magnetic state, the saturation magnetization (M_S_, the maximum magnetization recorded when all the magnetic moments in the sample are aligned [15]), the coercivity (or coercive field or force H_c_ that corresponds to the value of the applied magnetic field needed to demagnetize a magnetized material [310]), the uniaxial magneto-crystalline anisotropy energy constant [15] (K_u_), the blocking temperature (T_B_, characteristic of superparamagnetism [15]) and the maximum energy product ((BH)_max_) [142].

Table 5 shows experimental structural and magnetic properties of available MPt (M = Fe, Co) nanoparticles used as ORR catalysts; where possible, the above-mentioned parameters have been correlated with the catalytic activity via specific activity (SA) and Tafel slope (many studies provide catalytic activities, but lack fundamental magnetic characterization of the catalysts). Table 5 also indirectly indicates that combining theoretical concepts, catalytic procedures and electrochemistry into the synthesis of active magnetic compositions for relevant industrial chemical processes is a challenging task.

It should be clear by now that a synergistic approach to theory, computation and experiments is essential to find active compositions in the production of clean energy. The most active magnetic compositions in ORR and in other catalytic processes, such as OER [21,321,322,323] and HER [23], typically contain Fe, Co and Ni. Indeed, as described previously, Pt-Fe, Pt-Co and Pt-Ni alloys are considered the most acclaimed ORR catalytic materials. Moreover, these alloys and some of the Pt-free catalysts, such as phthalocyanines containing Fe (FePc) and Co (CoPc) atoms, are also under intensive research in the field of spintronics [134,267,324]. Spintronics, or spin-based electronics, is based on materials where the electron spin carries the information and not its charge [325,326]. Metals such as Fe, Co, Ni and their alloys also possess specific conductivity behaviors [327,328] described by the “two currents” concept proposed by N. F. Mott [72]. Mott’s ideas have been further elaborated by A. Fert and I. Campbell to elucidate several transport properties of FM *3d* metals and their alloys by assuming that spin up and spin down originate two conduction currents in parallel [328]. This is one of the reasons why these materials are exploited in spintronics devices [329] and heterogeneous catalysis [19]. Another reason is found in their peculiar magnetic properties. Fe, Co and Ni are indeed well-known ferromagnetic metals, and most of their alloys exhibit ferromagnetism (e.g., FePt and CoPt, both in their disordered and ordered phases). P. W. Selwood knew that FM components are needed to obtain active catalysts [258,330]. J. A. Hedvall tried to provide an explanation on the relationship between the magnetic properties of the catalyst and its catalytic activity by introducing the concept of the *internal magneto-catalytic effect* [293,294]. A more modern attempt to provide a missing link between catalytic properties and magnetism was provided by J. Gracia with the introduction of QSEIs [19,56,282]. Nevertheless, the relationship between ferromagnetism and catalytic activity is still an open quest. The main challenge in using FM compositions to catalyze reactions lies in matching the Curie temperature with the temperature range at which the catalytic process occurs. This means that in order to exploit the magnetic properties of FM compositions, the chemical reaction must occur below the Curie temperature of the catalyst, which in turn depends on several factors such as size, shape and preparation method, as shown in the cases of fct FePt and fct CoPt NPs in Table 4 and Table 5.

### 3.6. Application of External Magnetic Field ($\vec{H}$, Extrinsic Magnetism)

The application of an external magnetic field $\left(\vec{H}\right)$ was the first approach used to investigate the connection between magnetism and catalysis [258]. The investigation of the magnetic response of quantum (quasi)-particles (i.e., fermions) under an applied magnetic field in particle and nuclear physics is referred to as *magnetic catalysis* [331,332]. The effects of $\vec{H}$ on chemical reactions, also indicated as external MFEs (magnetic field effects), is a well-known and investigated phenomenon [258,333,334,335]. It potentially involves all paramagnetic compounds and materials since, as previously described, these materials are sensitive to the application of an external magnetic field. It follows that the catalytic activity of all catalysts based on paramagnetic species/metals may be modified by the presence of an external magnetic field [258,259].

The most famous and emblematic case in heterogeneous catalysis is the ortho-para hydrogen conversion onto (para)magnetic surfaces [258,335,336,337,338]. The external MFE generally observed is the variation in reaction rate [335,339], but some authors also reported a change in the conductivity of the solid catalyst [333]. For example, P. W. Selwood reported a postulated increment in the ortho-para H_2_ conversion rate for rare earth oxide Er_2_O_3_ as catalyst and a likely decrement for Pr_2_O_3_ at room temperature and under a weak external magnetic field [340,341]. A more marked effect was observed using ferromagnetic catalysts such as nickel [342], for which Selwood reported a great increment in the rate of ortho-para H_2_ conversion [340]. He also reported that the rising conversion rate was proportional to strength of the applied magnetic field and to the temperature [340], indicating that the effect was stronger above the T_C_ of the Ni catalyst [340] (T_C_ of Ni is 631 K) [15]. Selwood obtained similar results for α-Cr_2_O_3_ with temperature above the Néel temperature (T_N_ = 308 K) [339,340]. He explained this experimental result through the so-called *magneto-catalytic effect* [335,340]: the presence of an internal magnetic field due to the catalyst (previously mentioned *internal magneto-catalytic effect*) and/or an external one (*external magneto-catalytic effect*) affects the rate of a catalyzed reaction [272,340]. In the case of the ortho-para conversion of H_2_, ortho-H_2_ (nuclear spins are ↓↓ or ↑↑) and para-H_2_ (nuclear spins are ↑↓) behave differently in the presence of an applied field. The conversion only happens when there is a realignment of the nuclear spins, which can occur without breaking the H-H bond (the so-called non-dissociative mechanism [335,340]) by using magnetic surfaces [335,336,340,342] or paramagnetic species such as ^3^O_2_ [343]. The application of an external magnetic field simply increases the rate of the nuclear spin realignment, which is generally called “field acceleration” [339,344]. Theoretical explanations of this “field acceleration” on (para-)magnetic catalysts were also provided by several authors [48,49,335,336,344,345,346].

The main scientific finding from the investigation of this reaction is that external magnetic fields can change the reactivity of reactions catalyzed by magnetic solids and that the magnitude of such effect depends on several factors, such as the catalyst surface electronic structure [340,341,344], the type of the reaction [258,333], the temperature (as seen by Selwood when using Ni as catalyst in the H_2_ conversion [340]), the strength [340,341] and the orientation [347] of the applied magnetic field. Moreover, these conclusions were and are still not only restricted to ortho-para H_2_ conversion, but can be applied to all the heterogenous processes where the adsorbed reagents react with the catalyst surface (i.e., with the spins of the material) through magnetic interactions [258]. Indeed, if an applied magnetic field is able to influence the reactivity of the ortho-para H_2_ conversion by interacting with nuclear spins, then the effect is expected to be stronger when electron spins are involved [333].

Despite such evidence, separating, discriminating and quantifying changes only due to the application of the external magnetic field and not to other parameters (e.g., thermal effects, the influence of the intrinsic magnetic properties of the catalyst, among others) during catalytic operations are extremely challenging, especially when dealing with solid-state catalysts [335,348]. Nevertheless, the topic is still attracting a lot of attention in the scientific community as a tool for promoting and boosting catalytic processes involved in the production of clean energy [348,349]. *Magnetoelectrochemistry* is the discipline devoted to unifying electrocatalysis and magnetic phenomena derived from the application of an external magnetic field (in constant or alternating mode) [348,350,351]. Several magnetic forces (e.g., Lorentz force, Kevin force, field gradient force, paramagnetic force) and magnetic phenomena (e.g., magnetoconvection phenomena) [348,349,350,352,353,354,355,356,357,358] take place in an electrochemical cell subjected to an applied external magnetic field. The resulting effects are typically divided into three groups:

- Changes in the mass transport;
- Modification of the heterogeneous electron transfer kinetics and electrochemical equilibria; and
- Influence on electrodeposit morphology.

Several experiments have confirmed an improved mass transport, an improved H^+^ transport from the electrolyte to the cathode surface, a faster desorption of H_2_ bubbles at the electrode surface and a stabilization of the electrochemical membrane upon application of an external magnetic field [348,349,359]. Other experiments show that the application of a constant magnetic field enhances the resistance of several coatings against corrosion and generates smoother surfaces with finer grains when metals and alloys are prepared as coating materials [359,360,361]. Even though *magnetoelectrochemistry* is a mature discipline after more than twenty years and the effects on the mass transport and on the deposit morphology are well known and established [358,359], the possible influence of the external magnetic field on the electrode kinetics and on electrochemical equilibrium remains unclear and under debate [348]. Unfortunately, no experimental or theoretical evidence points unanimously toward the existence of this effect [348,358,359].

### 3.7. Combination of Intrinsic and Extrinsic Magnetism in Catalysis ($\vec{H_{0}}+\vec{H}$)

The combination of the two previous effects (intrinsic + extrinsic magnetism) represents the most promising way to boost catalytic processes nowadays [348,362]. There are numerous examples in the literature showing that magnetic compositions containing Fe, Co and Ni exhibit outstanding catalytic performances when an external magnetic field is applied. Some of these examples are found in ORR [363,364,365,366,367], OER [368,369,370], HER [371,372,373,374] and water splitting reactions [288]. The same outcome is also observed in devices such as lithium-based batteries [362,375,376].

The use of ferromagnetic compositions is justified by their stronger response to the presence of an applied magnetic field in comparison with diamagnetic or paramagnetic materials [15,258]. J. M. D. Coey and co-workers investigated the ORR catalytic activity of metal particles of Fe, Co and Zn with similar shape and size under the presence of a magnetic field of 360 mT, generated behind the cathode [367]. They obtained an increased maximum oxygen reduction current of 11.6 ± 1.8% and 7.8 ± 1.2% for FM Fe and Co particles, respectively, while only a 3 ± 0.7% increment was seen for diamagnetic Zn metal particles under the same conditions [367]. Coey and his group also carried out several more works dedicated to the role of magnetic fields in ORR [377,378]. Moreover, different authors pointed out that some external magnetic field effects (MFEs) in electrocatalysis are more marked or can only be observed when ferromagnetic materials are employed [258,352,353,354,356,360]. For example, some of them reported that both Lorentz and Kelvin forces are involved when an FM material is employed as ORR catalyst in the presence of an applied field, while only the former is implicated if the catalyst is nonmagnetic (i.e., closed-shell composition with no spontaneous magnetization) [366,367].

Table 6 summarizes scientific works reported in the literature and featuring the use of external magnetic fields and/or magnetic Pt-based catalysts to boost/improve ORR. Though the catalytic improvements of ORR catalysis via the synergistic combination of magnetic catalysts and external magnetic fields seem to be a well-documented reality, the investigation and the understanding of how catalytic performances of magnetic catalysts (especially FM compositions) are further enhanced under an applied external magnetic field still need to be fully addressed.

Several other examples regarding the effects of magnetic fields in ORR when catalysts are different from Pt and/or its alloys also available in the literature [363,365,366,380,381,382,383,384].

**Figure 16:**
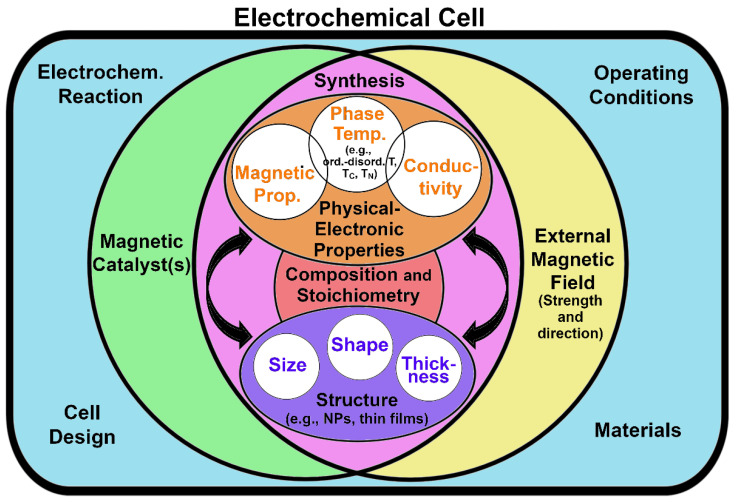
The most important factors and their interdependency in the magnetism-catalysis relationship for clean energy production.

Cutting-edge knowledge from several different and diverse fields is involved in exploiting magnetism in catalytic reactions. Figure 16 summarizes the most important parameters and their interdependencies to design and synthetize active compositions for electrochemical reactions, such as ORR, in order to exploit them in modern technological devices for the production of clean energy.

## 4. Basics of Fuel Cells

### 4.1. Renewable Energy Demand and Energy Storage Systems

Low-carbon energy technologies represent the future of clean energy sources nowadays [1]. Their importance is rising day by day due to the growing demand for industrial electricity and the increased awareness worldwide of the need to reach a net-zero emissions scenario [385]. Thus, the development of innovative energy storage systems (ESSs) has become of crucial importance to help meet these industrial and environmental goals. Different types of ESSs are employed: mechanical (e.g., compressed air energy storage and pumped hydroelectric storage), chemical (e.g., hydrogen storage with fuel cell), electrochemical (e.g., different types of batteries and fuels cells), electrical (e.g., super-capacitors and superconducting magnetic energy storage) and thermal systems (e.g., latent heat storage) [386]. Among them, electrochemical energy storage systems stand out for their efficiency, accessibility, reliability and as user-friendly devices with a wide range of applications.

### 4.2. Electrochemical Energy Storage Systems: Batteries and Fuel Cells

Five types of electrochemical ESSs are commercially available: primary batteries, secondary batteries, battery systems for grid-scale energy, fuel cells and electrochemical capacitors (see Table 7). Among these five ESS types, fuel cells emerge as the most environmental friendly solution [1,10]. Nonetheless, batteries may still contain pollutants, such as heavy metals (e.g., Pb, Cd, Hg, Cr and V), which should be properly collected, treated, recycled and buried in order to reduce severe environmental concerns and public health issues [386].

The environmental compatibility of fuel cells is just one of various potential advantages of this technology. The high thermodynamic efficiency and the possibility to co-generate electricity and heat are attractive features. Fuel cells, in fact, hold great promise for several domestic and industrial applications [1,385]; for instance, fuel cells can be employed as a combined heat and power system to provide energy in hospitals and other public buildings, as auxiliary power units (APUs) in vehicles, as energy sources for portable devices and, above all, as propulsion systems in electric (FCEVs) and hybrid electric vehicles (FCHEVs) [3,5,387,388].

Fuel cells, however, also possess a few disadvantages. One of the main issues concerns the high production costs that are linked to the use of expensive raw materials, such as scarce noble metals (e.g., platinum, Pt) [10]. Another non-negligible concern is the use of gaseous hydrogen (H_2_) as fuel—storage space and safety become serious challenges for large-scale commercialization [10,389]. A SWOT (Strengths, Weakness, Opportunities and Threats) analysis is reported in Table 8 to provide an overview of the strengths and weaknesses of this technology.

Despite the inherent weaknesses and challenges emerging from the SWOT analysis, the International Energy Agency (IEA) and the European Union identified hydrogen and fuel cells as cross-sectorial solutions to fight CO_2_ emissions and hydrocarbon dependency and to improve economic growth [385,387]. For instance, the implementation of fuel cells in electric and hybrid vehicles meet the IEA and European commitment for a future based on a low-carbon economy [1,387]. Moreover, the issues in fuel cells are far from insuperable. A few solutions regarding the storage and the transport of hydrogen are currently available and under development, such as the use of high-pressure cylinders, cryogenic liquid hydrogen or metal adsorbers/“hydrogen carriers”, which generate the gas in situ [10,389]. Environmental concerns and economic interests have recently boosted research [390] and investment in fuel cell technology and hydrogen (H_2_) exploitation [387,389]. The aim is to break the confinement of this groundbreaking technology to research laboratories and facilitate its entry into the market.

### 4.3. Fuel Cells

A fuel cell is a reaction chamber, composed of two electrodes (cathode and anode) and an electrolyte, where an electrochemical process generates electricity and power as long as the fuel is provided, since no relevant chemicals are present inside the cell. The external provision of the starting materials is the primary difference between a fuel cell and a battery. The external provision of fuel allows the uninterrupted production of electrical currents.

The electrochemical process that takes place in a fuel cell is the direct formation of water (H_2_O) from hydrogen (H_2_) and triplet-state oxygen (^3^O_2_) gases (2H_2(g)_ + ^3^O_2(g)_ → 2H_2_O). This direct oxidation of the fuel (H_2_/^3^O_2_ gases) is one of the features of this promising technology that makes it environmentally attractive. Although this reaction produces only water as a by-product [3,385], it is nonetheless a kinetically slow reaction at operating temperature and it needs a proper catalyst(s) to be activated [3,10].

**Figure 17:**
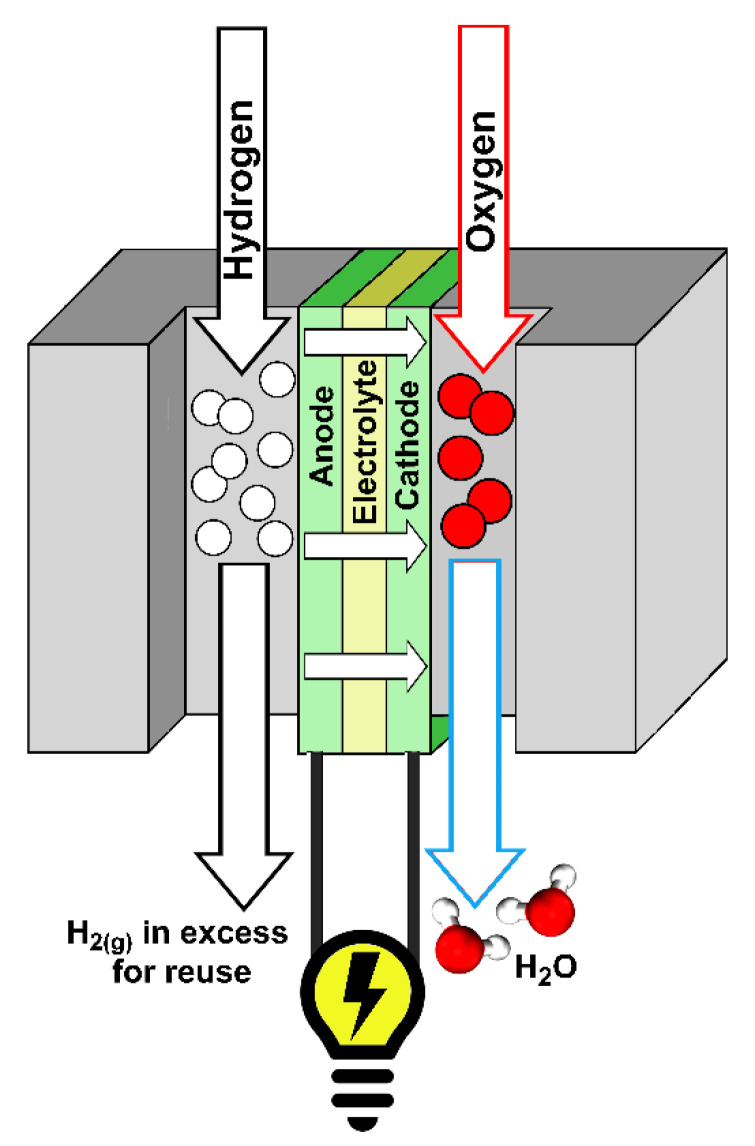
Simple scheme of the electrolytic cell used in fuel cells.

**Figure 18:**
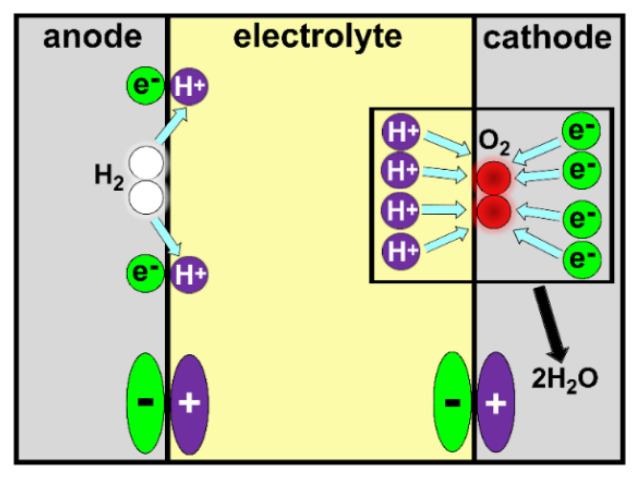
Sketch of the electrochemical process in a fuel cell when an acidic solution is used as electrolyte.

The formation of water is split into two electrochemical reactions: the oxidation of hydrogen (hydrogen oxidation reaction, HOR) at the anode and the reduction of oxygen (oxygen reduction reaction, ORR) at the cathode (see Figure 17). The proton exchange is guaranteed by an electrolytic solution that can be acidic or alkaline. In the case of an acidic solution (the most used conditions in PEMFC) [3,10], the hydrogen gas delivered at the anode is ionized and H^+^ ions (protons), electrons (e^−^) and energy are released (2H_2(g)_ → 4H^+^ + 4e^−^, HOR). Concomitantly at the cathode, the gaseous oxygen reacts with the electrons of the electrode and H^+^ ions to generate water (^3^O_2(g)_ + 4e^−^ + 4H^+^ → 2H_2_O, ORR). Figure 18 shows a detailed scheme of both half-cells during this electrochemical process.

As seen at a glance, the breaking of two hydrogen molecules at the anode (only one is shown in Figure 18 for clarity) releases four protons (H^+^) and four electrons (e^−^). The released protons migrate into the electrolyte, while the four electrons remain on the electrode surface, which becomes negatively charged. At the same time, one molecule of oxygen arrives at the cathode and acquires the previously released four protons from the electrolyte and the four electrons from the metal of the cathode. These events make the electrolyte solution negatively charged and the metal surface of the cathode positively charged. The outcome of the overall reaction is two molecules of water [391]. The acidic electrolyte transports H^+^ ions from the anode to the cathode and prevents the transport of the electrons, which move instead through an external electric circuit (Figure 17). This configuration allows the process to run continuously [10].

On the contrary, OH^−^ ions (hydroxyl ions) are transported in an alkaline electrolyte; a different pathway is active in this case. The hydrogen at the anode is oxidized to produce water, releasing four electrons and energy (2H_2(g)_ + 4OH^−^ → 4H_2_O + 4e^−^, HOR). At the same time, the reduction of the oxygen (ORR) consumes the water and the four electrons from the cathode to release new OH^-^ ions into the electrolyte (^3^O_2(g)_ + 4e^−^ +2H_2_O → 4OH^−^, ORR). Again, an external electric circuit is needed to allow the delivery of electrons from the anode to the cathode. Nonetheless, the overall reaction, 2H_2(g)_ + ^3^O_2(g)_ → 2H_2_O, remains unchanged. The only difference between a process employing an acidic electrolyte and one employing an alkaline electrolyte is the site of the water production, which takes place at the cathode or the anode, respectively.

### 4.4. Thermodynamics of Fuel Cell

The simple electrochemical cell shown in Figure 18 represents a so-called open circuit since no external electrical apparatus is connected to the system; the corresponding voltage, generated between the anode/electrolyte and the cathode/electrolyte interfaces, is known as open circuit voltage (OCV) [10,391]. In a true fuel cell, as depicted in Figure 17, however, an external circuit is also present. The flow of electrons passing through it is essentially an electrical current, and its transport comes with a cost since the system has to carry out external work. The available energy required to provide such external work corresponds to the Gibbs free energy (∆G) [10,391,392]. Thus, fuel cells can be defined as open thermodynamic systems where ∆G is directly transformed into electrical energy. When the system is assumed to be reversible (thermodynamically in equilibrium), the relationship between open circuit voltage (OCV) and Gibbs free energy is a direct proportionality, as described by Equation (28) [10,392].

(28) $$E=\left(-\Delta G\right)/\left(n_{e}F\right)$$

where *E* is the reversible OCV of the cell, ΔG is the Gibbs free energy, *n_e_* is the number of electrons transferred per mole of fuel and *F* is the Faraday constant (96,485.332 C/mol) [10,392]. As a generalization, the Nernst equation describes the OCV via the relationship between the voltage and the species concentrations both far from and at the equilibrium [10].

The definition of the open circuit voltage is key to understanding the operating system of a fuel cell since it provides a theoretical value of the voltage produced when no voltage loss is present (ideal fuel cell). As a consequence of Equation (28), parameters that affect ΔG, such as temperature, pressure and concentrations (better yet, activities) of the reactants, also affect the voltage [10]. In a real fuel cell, the effective operating voltage (experimental value) is smaller than the OCV (theoretical value) since the process is not completely reversible and voltage losses inevitably occur. Such discrepancy is commonly defined as overvoltage or overpotential [10] in electrochemistry and can be caused by various factors called “irreversibilities” or simply losses [10]. The most relevant are:

- Activation losses, due to kinetics of the electrochemical reaction at the electrodes. A part of the voltage is used to drive the electron transfer from one electrode to the other during the electrochemical reaction (major voltage loss).
- Fuel crossover and internal currents, caused by incomplete fuel utilization. The majority of the fuel reacts, but a small amount diffuses through the electrolyte unused (this loss increases in fuel cells operating at low temperatures).
- Ohmic losses, due to the electrical resistance of the material of the electrodes, the electrolyte solution and other components of the fuel cell.
- Mass transport or concentration losses, connected with the consumption of reactants at the electrode surface, that cause a change in their concentrations (or, more precisely, activities), thus to the voltage.

The maximum efficiency of a fuel cell lies at the maximum value of OCV, and its efficiency is the measure of the amount of released electricity: the more electricity released at a constant fuel quantity, the more efficient the fuel cell. Such a concept is valid both for ideal and real processes, and the real efficiency of a fuel cell is always less than the ideal one, as in the case examined for operating voltage. This is partially due to the voltage drops, but also to other issues generally related to a suboptimal fuel utilization [10,392].

### 4.5. Kinetics of Fuel Cell

The performance of a fuel cell does not depend only on the amount of chemical energy transformed into electric (i.e., thermodynamics), but also on the rate of this transformation (i.e., kinetics). Indeed, the electrochemical reactions occurring at the anode (HOR) and cathode (ORR) happen at different rates. The electrochemical transformation in an FC involves the transfer of electric charges at the boundary between the electrodes and the electrolyte and, concomitantly to this internal charge transfer, an electron movement along the external circuit (Figure 17). The electric current (i) is defined as the measure of the number of electrons flowing during a certain time gap in this external circuit. The quantity of current obtained during the electrochemical reaction in a fuel cell provides the rate of the internal charge transfer, which is expressed with finite values [392].

We mentioned before that the formation of water (2H_2(g)_ + ^3^O_2(g)_ → 2H_2_O) must be activated by a catalyst. The role of the catalyst is to lower the activation energy by providing a faster alternative pathway to the process. The activation energy represents the energy barrier to overcome in order to pass from one side (i.e., reactants/products) to the other side (i.e., products/reactants) of the reaction. In a fuel cell, this usually means the consumption of some voltage and consequently the generation of activation loss or, more commonly, activation overvoltage ($\Delta V_{act}$). $\Delta V_{act}$ is strongly correlated with the ability of a catalyst to lower the activation energy of the electrochemical process. Two equivalent approaches are used to define $\Delta V_{act}$: the Butler–Volmer (Equation (29)) and the Tafel equation (Equation (30)). The Butler–Volmer equation is [10]:

(29) $$j=j_{0}exp\left(\frac{z\alpha F\Delta V_{act}}{RT}\right)$$

where:

- j is the current density, the current per unit area (A/cm^2^) (a more important parameter than the simple current, since the reaction takes place at the electrode/electrolyte interface);
- $j_{0}\;$is known as exchange current density;
- $\Delta V_{act}\;$is the activation overpotential;
- α is the dimensionless charge transfer coefficient that corresponds to the quantity of the electrical energy used to modify the reaction rate at the anode and cathode (its value depends on the type of electrochemical reaction and electrode material, but it ranges from 0 to 1.0) [10];
- z is the number of electrons involved in the electrochemical process;
- R is the universal gas constant;
- T is the absolute temperature; and
- F is the faraday constant.

The Tafel Equation (30) is [10]:

(30) $$\Delta V_{act}=A\cdot \Delta ln\left(\frac{j}{j_{0}}\right)$$

where $\Delta V_{act}$ is the activation loss, A is the charge transfer coefficient for the electrodes (a constant connected to α in a simple way, since A=(RT)/(zαF) [10]) and, again, j and $j_{0}$ are the current density and the exchange current density, respectively. Equation (30) represents a way to express the Tafel equation that is derived from experimental evidence [10], unlike the Butler–Volmer equation.

The meaning of the Butler–Volmer Equation (29) is that the current produced by an electrochemical reaction increases exponentially with the activation overvoltage [392]. This means that a certain amount of voltage must be sacrificed to generate more electric currents in a fuel cell. The same concept is expressed by the Tafel Equation (30), but in another form: larger A values and a small exchange current density ($j_{0}$) indicate an increment in the activation overvoltage, typical of slow reactions [10]. The most important parameter in Equations (29) and (30) is represented by the exchange current density ($j_{0}$), the value of current density necessary to establish equilibrium between reagents and products [10,392]. Usually, higher values of $j_{0}$ are always desired in order to minimize the activation voltage loss (ΔV_act_) [10]. $j_{0}$ can be modulated by changing the metal used as a catalyst; this indicates a strong catalytic effect [10]. The catalyst, essential for the electrochemical process, increases the rate of the chemical transformation affecting the exchange current density (Equations (29) and (30)) and thus also the performance of the fuel cell. It goes unsaid that the research for an effective catalyst to reduce the activation overpotential is one of the major concerns in fuel cell development. This is especially true at the cathode of a fuel cell, where $j_{0}$ is much smaller than at the anode—$j_{0}$ at the cathode can sometimes be 10^5^ times smaller than the corresponding value at the anode [10]. In other words, the oxidation of hydrogen at the anode occurs six or more orders of magnitude faster than the corresponding reduction of oxygen at the cathode [6]. Nevertheless, the use of an optimal catalyst is not the only way to reduce the activation overpotential. Other ways to minimize $\Delta V_{act}$ are known: raising the cell temperature, increasing the roughness of the electrodes (thus the number of possible reaction sites) and increasing the concentration of the reactants (e.g., by using pure hydrogen and oxygen gasses) [10].

In summary, the reduction in the activation overvoltage still represents a challenge directly related to the fuel cell electrochemical process.

### 4.6. Types of Fuel Cell

Various categories of fuel cells exist at present [3,393]. Each possesses different engineering characteristics, specific electrolytes and distinct fuel requirements that make them exploitable in various applications. These types are:

- Alkaline Fuel Cells (AFCs): They use an alkaline liquid (K_2_CO_3_ or KOH) as electrolyte. The first models operated at high temperatures (50–200 °C) [6], but AFCs can operate at lower temperature (20–80 °C) nowadays [3]. No high-profile research on these FCs is currently ongoing, due to their higher capital cost compared to the other fuel cell categories, but they were exploited in the 1960s for space programs with a great deal of success [6].
- Phosphoric Acid Fuel Cells (PAFCs): They work at high temperatures (~220 °C) and use an inorganic acid (100% concentrate phosphoric acid) as proton-conducting electrolyte [6]. These were the first examples of commercially available fuel cells, thanks to their reliability as a power source, durability and low maintenance [6]. PAFCs are exploited in power stations nowadays [3].
- Molten Carbonate Fuel Cells (MCFCs): They also operate at high temperatures (600–700 °C), use a molten mixture of alkaline metal carbonate (lithium and potassium or lithium and sodium carbonate) as an electrolyte ((CO_3_)^2−^ is the mobile ion) [6] and, unlike AFCs and PACFs, exploit abundant metals as catalysts (nickel and nickel oxides). MCFCs display severe corrosion and stability issues at present, making them unappealing for the market [6].
- Solid Oxide Fuel Cells (SOFCs): They are solid-state devices composed of a solid and a gas phase. The anode contains ceramic zirconia cermet with nickel metal and the cathode contains a mixture of electronically conducting ions and ceramics (for example, strontium-doped lanthanum manganite) [3,10]. SOFCs are currently working at high temperatures (600–1000 °C) [3] and are still under development [10].
- Proton Exchange Membrane Fuel Cells (PEMFCs): They operate at low temperatures [10] (−40–90 °C) [3] and are currently the most quoted type, since they embody the most promising solution to address environmental concerns worldwide [3,6,394,395].

All types of fuel cells previously described are based on inorganic catalysts, Pt-based materials above all. There is, however, another kind of fuel cell that exploits organic molecules, especially enzymes, as catalysts. Such fuel cells are generally called biological fuel cells [10,165], and two possible implementations are now under study: enzymatic fuel cells, which directly use the enzymes as catalysts, and the microbial fuel cells, which use the enzymes contained in microorganisms. The fuel in these systems is composed of organic fuels such as methanol and ethanol. Currently, there are no commercial applications for biological fuel cells, even though they are carefully investigated as energy storage systems in implantable medical devices, due to their small size (0.07 cm^2^ in area) and the low amount of electric current generated (estimated at about 300 μV for 2 h) [396].

### 4.7. Proton Exchange Membrane Fuel Cells (PEMFCs)

Proton exchange membrane fuel cells (PEMFCs) were the first fuel cell type, together with alkaline fuel cells (AFCs), used in space missions since the 1960s. Unlike AFCs, however, PEMFCs still attract a lot of attention as innovative energy storage systems. PEMFCs are actually the most investigated fuel cells nowadays and possess a wide range of applications from small and medium-sized devices (e.g., mobile phones and laptops) to large-scale systems (e.g., combined heat and power systems). The most appealing application for PEMFCs is in the automotive sector [1,3,5,6].

#### 4.7.1. PEMFCs Components

**Figure 19:**
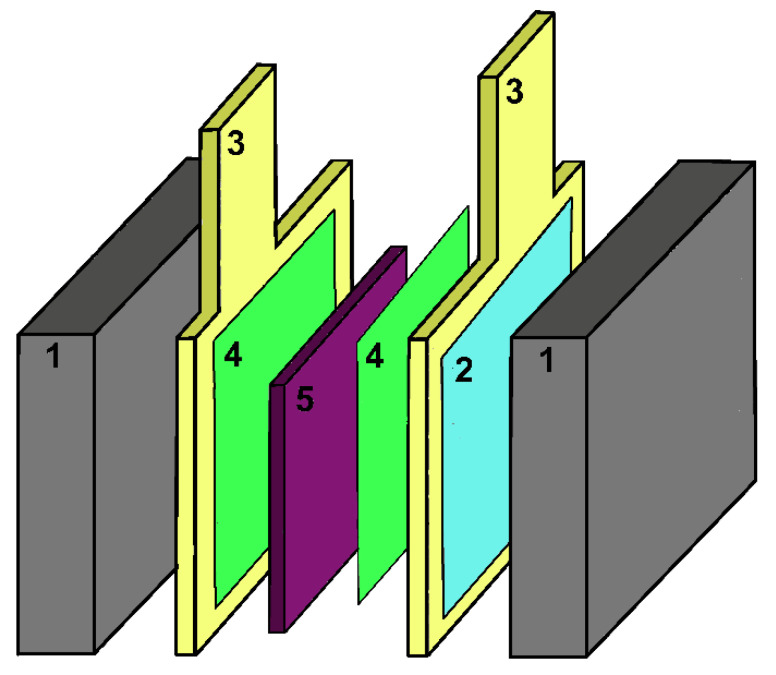
Scheme of the components in PEM fuel cells: bipolar plates (1), current collectors to prevent gas leakage (2), gaskets (3), gas diffusion layer (GDL) (4), membrane electrode assembly (MEA) (5).

Figure 19 shows the common design and the typical components used to build a single PEMFC [2,3,5,393,397,398]. Bipolar plates (component **1** in Figure 19) allow the conduction of electric currents from the anode of one cell to the cathode of the next one, the distribution of the fuel gas over the anode surface and the oxygen over the cathode one, and the management of water and heat by transporting cooling fluids [3,398]. The choice of the bipolar plate material is crucial and relies on various required chemical and physical properties, such as hydrogen permeability, corrosion resistance (due to the contact with the acid electrolyte, oxygen, hydrogen, heat and humidity), electrical conductivity, thermal conductivity, cost and weight [5,10,399]. The common investigated materials are non-porous graphite/electrographite, metals (coated and non-coated) and polymer–carbon materials [3,400]. The gas diffusion layer (GDL, component **4**), shown in Figure 19, is a thick carbon-based layer that support porous electrodes, protects the catalyst against corrosion and degradation, allows the diffusion of the gases toward the catalyst and enhances the electrical conductivity [3,5,401]. The most important compartment of a PEMFC is the anode–electrolyte–cathode assembly, usually called membrane electrode assembly (MEA) (component **5** in Figure 19). The MEA unit represents the electrochemical work station of the fuel cell [2,3,5,10]. The electrolyte is composed of a solid polymer ion exchange membrane (commonly sulphonated fluoropolymers) [3,10,402]. The electrodes are generally made of a porous and conductive material (e.g., carbon cloth or carbon paper) on which the catalyst is anchored through specific techniques [3,402,403]. The careful design and choice of materials are again required to reach high performances and durability in the electrodes [5,402,404,405,406,407]. For example, carbon supports have gained a lot of attention in recent years, thanks to their critical role in the kinetics of ORR, in the transport of ^3^O_2_ and in the loss of the electrochemically active surface area (ECSA) of the catalyst [5,400]. At present, the state-of-the-art commercially available MEA exploited in FCEVs (e.g., Toyota Mirai) [24] uses electrodes supported on a high-surface-area carbon material and composed of platinum metal (Pt) and platinum–cobalt (Pt-Co) alloy at the anode and cathode, respectively [5].

#### 4.7.2. Applications of Magnetic Field in PEMFCs

Several factors affect the performance of PEM fuel cells, such as the preparation method of the catalysts layer [408,409], the design of catalyst ink [410], the catalyst loading [411], the size of the catalyst [412], the flow field design to supply the fuels [413], the type of fuels [395], the presence of contaminations in the fuels [414], the choice of materials [401,415], the operating conditions [395,401,416] and so on. Additional parameters that can affect the performance of a PEMFC are magnetic fields.

The implementation of magnetic fields to improve the catalytic performance of PEM fuel cells represents an innovation in the field. The magnetic fields can be generated externally (applied magnetic field) or internally (magnetic catalyst) to the cell. Generally, when an external magnetic field is applied to a PEMFC, the following effects have been experimentally documented: a modified mass transfer rate of ^3^O_2_ and H_2_ gasses, an improved activation of these molecules [364,381,397] and an overall enhancement of the fuel cell performance [381,397,417,418] also at low temperature [418]. Similar effects are reported when the applied magnetic field is employed to magnetize the catalyst or the MEA of the PEM fuel cell. Table 9 summarizes the experimental observations seen in operating PEMFCs (single or stack experiments), using Pt or Pt-M alloys as a catalyst, in the presence of an external magnetic field or magnetic catalysts.

Magnetic fields can also be used to produce magnetized ionomer membranes which commonly lead to an improvement of the PEMFC performance. J. Tang and co-workers prepared a magnetized perfluorosulfonate ionomer (PFSI) membrane with a through-plane orientation via induction of magnetic field [422]. The group observed improved PEMFC performances and lower cell resistance, especially under low humidity (T~323, 353, 343 K), when using magnetized vs. non-magnetized PFSI (at equal conditions); the group also observed decreased hydrogen permeability [422]. Another example of magnetized composite is the phosphotungstic acid (PWA) combined with proton-conducting polymer membrane (CP4VP), prepared by X. Liu and colleagues via application of a strong magnetic field [417]. The composite membrane exhibited high proton conductivity, better efficiency and longer durability in single fuel cell experiments (commercial Pt/C as catalyst with 0.24 mgPt/cm^2^) [417]. Such improvement in efficiency is attributed to the formation of oriented mass transfer channels due to the alignment of the through-plane proton-conducting channels in the membrane, induced by the applied field. Moreover, the high durability of the membrane is attributed to the fact that the magnetic field effect promotes the formation of a more stable paramagnetic PWA-CP4VP complex [417]. The use of strong magnetic fields was also employed by L. Liu and co-workers to prepare magnetized Nafion composite membranes with sulfonated graphene oxides (SGOs) [423]. The group observed that magnetizing just 1% in weight of SGOs was enough to achieve superior performance for proton transport (~37% of enhancement) in comparison with non-magnetized membranes in single fuel cell tests (T = 333 K). Similar results were also obtained by J. Hyun and co-workers; the group prepared proton-conductive paramagnetic and one-dimensional tungsten disulfide (WS2) nanotubes as components of a perfluorinated sulfonic acid (PFSA) membrane [424]. The magnetization of the nanotubes by using a weak TP magnetic field of 0.035 T induced the formation of an aligned PFSA membrane. Such magnetized membrane showed higher proton conductivity (~69%) than a normal Nafion membrane with the same thickness [424]. A. M. Baker and co-workers magnetized Fe_3_O_4_-MWCNT (multiwall carbon nanotube) nanoparticles with a plate magnet to prepare composite membrane containing Nafion with an improved ~45% tensile strength. They also reported an improvement in the fuel cell performance in comparison with a non-magnetized Fe_3_O_4_-MWCNT-based composite membrane under equal conditions [425].

Another use of magnetic fields in PEMFC is to employ them to enrich the content of oxygen in the atmospheric air used as fuel [426]. Electromagnetic air pumps are devices that integrate magnetic fields within air pumps that can be exploited in portable PEMFCs [427,428]. Magnetic fields are also self-generated by the same PEMFC. Mapping modifications of such magnetic fields can be employed as non-invasive diagnosis methods to detect and isolate faults in PEMFC themselves. Changes in current density distribution inside PEM and overall performances can be monitored using magneto-tomography data [429,430,431,432,433,434,435,436]. Some authors also investigated the effect of these self-induced magnetic fields on the corrosion of the metal bipolar plates in PEMFCs. S. Feng and co-workers investigated such effects on Ti- and Au-coated Ti (Ti/Au) cathode plates [437]. The self-induced magnetic field was simulated by inserting a Nd-Fe-B permanent magnet into the electrodes. Durability tests on the cathode environment, carried out in 0.0005 M H_2_SO_4_ and 0.1 ppm HF as electrolyte at 353 K, revealed that Ti and Au-coated Ti bipolar plates showed a lower degree of corrosion when a self-induced magnetic field was generated under operating conditions. Such results may suggest that magnetic fields, generated by the operation currents in PEMFCs, could extend the lifetime of bipolar plates, especially when Ti/Au is employed. The authors also pointed out that the effects of external magnetic fields on the service life of metal bipolar plates have not been investigated in real fuel cells yet. In fact, studies on the possible corrosion effects due to the application of magnetic fields are limited to metals such as Fe, Zn and Al [438,439,440] and to permanent magnets, such as Nd-Fe-B and Nd-Fe-Cu-B [441,442,443], in both acidic and neutral solutions.

Several patents regarding the implementation of magnetic fields in PEM fuel cells have been registered in the past three decades [444,445,446,447,448].

## 5. Conclusions

Table 10 offers a summary of the reported experimental effects observed in ORR when using magnetic catalysts, the application of an external magnetic field and the combination of these two strategies.

Magnetism in catalysis is a “hot” topic in all chemical reactions where magnetic, paramagnetic or, more generally, substrates and/or intermediates with unpaired electrons play a significant role, as in the case of ^3^O_2_. The comprehension of the complex relationship between magnetism and heterogenous catalysis for reactions with industrial importance, such as oxygen reduction reaction (ORR), is still an interesting yet intricate and incomplete quest. An improved comprehension will help in devising and/or improving novel synergistic strategies to design, synthesize and build efficient electrochemical devices for the production of clean energy for a more sustainable future, one of the most challenging issues worldwide.

This review is meant as a straightforward and approachable guide to provide a congruous, well-organized and interconnected (also from a historical point of view) package of information on the topic of magnetism, heterogeneous catalysis, Pt_x_M_y_ (M = Fe, Co, Ni) alloys and proton exchange membrane fuel cells (PEMFCs). This work presents a thorough description of structural aspects of known intermetallic PtxMy alloys and their links to magnetic properties and, consequently, catalytic activity in ORR. The improvement in the thermodynamics and kinetics of such reactions is crucial to achieve outstanding performances in the most prominent devices, such as PEM fuel cells.

Essentially faithful to the “golden” rule on the existence of a strong correlation between electronic structure and activity being at the base of modern approach in catalysis, this work proposes the description of crucial aspects of magnetism and magnetic cooperative interplaying, spanning from pure theoretical notions to more experimental and applied insights, relevant to catalytic activity and applications.

## Figures and Tables

**Table 1:** Experimental NP size (nm), cell parameters (nm) and *c*/*a* ratio for intermetallic MPt (M = Fe, Co and Ni) catalysts.

System	NPs Size (nm)	Cell Parameters (nm)		*c*/*a* Ratio	Reference
		a	c		
**FePt**					
fcc FePt	-	0.3884	0.3884	1	Malheiro [116]
fct FePt	6.5 ± 0.3	0.3848	0.3724	0.96	Xiong [117]
fct FePt	~6.1	0.27069	0.3709	1.37	Chen [118]
fct FePt	~6.1	0.27248	0.37312	1.37	Chen [118]
**CoPt**					
fcc CoPt	2.5 ± 0.2	0.3844	0.3844	1	Loukrakpam [119]
fcc CoPt	~6.2	0.3803	0.3803	1	Watanabe [120]
fcc CoPt	2.5 ± 0.1	0.3797	0.3797	1	Oezaslan [121]
fcc CoPt	2.7	0.38732	0.38732	1	Travitsky [122]
fct CoPt	12.4 ± 1.4	0.3814	0.3704	0.97	Oezaslan [121]
fct CoPt	~6.2	0.2692	0.3662	1.36	Watanabe [120]
fct CoPt	~6	0.3780	0.3705	0.98	Xiong [117]
**NiPt**					
fcc NiPt	4.7	0.3821	0.3821	1	Xiong [117]
fcc NiPt	4.8 ± 0.5	0.3817	0.3817	1	Loukrakpam [119]
fcc NiPt	2.2	0.38486	0.38486	1	Travitsky [122]
fcc NiPt	3.2	0.38204	0.38204	1	Travitsky [122]
fcc NiPt	6.1	0.37368	0.37368	1	Carpenter [123]

**Table 3:** Reported ORR catalytic performances of MPt (M = Fe, Co and Ni) NPs prepared by different methods in comparison with commercially available Pt catalysts. All the electrochemical measurements, half-wave potentials ($E_{1/2}$), specific activity (SA), mass activity (MA) and Tafel slope were carried out in 0.1 M HClO_4_ solution at room temperature, unless otherwise specified. SA and MA are measured at 0.9 V vs. RHE, unless otherwise specified.

Entry	NPs Size (nm)	SA (mA·cm^−2^)	$\mathrm{MA}\;(A\cdot mg_{Pt}^{-1})$	Tafel Slope (mV·dec^−1^)	$E_{1/2}\;(V)$	Reference
**Pt**						
Commercial Pt/C	2–3	0.22	0.12	75	0.883	Li [243]
Commercial Pt/C	-	0.28	0.13	-	-	Li [246]
Commercial Pt/C	2.5–3.5	0.264 ^a^	-	-	0.531	Zhang [247]
Commercial Pt/C	3.2	-	-	-	0.905	Liu [248]
Commercial Pt/C	-	0.177	0.102	-	0.864	Ying [249]
Pt/C	~2.7	0.07	-	68.9	0.818	Du [250]
Pt/C	-	0.22	0.14	-	-	Chung [244]
Pt/C	<7.1 ± 1.6 ^b^	0.20	0.11	-	-	Loukrakpam [119]
Pt/C	-	1.70 ^c^	-	-	-	Gong [251]
Pt black	-	0.221	0.042	-	0.868	Ying [249]
**FePt**						
fcc FePt/C	8.5 ± 0.5	0.89 ^a^	-	-	0.533	Zhang [247]
fcc FePt/C	<10	-	-	-	0.890	Li [246]
fcc FePt/C	2.6 ^c^	3.95	-	-	-	Gong [251]
fcc FePt/CNT	2–3	-	-	-	0.894	Liu [248]
fct FePt/C	~6.1	0.578	0.272	-	-	Chen [118]
fct FePt/C	8.5 ± 0.5	2.1 ^a^	-	-	0.562	Zhang [247]
fct FePt/C	8.8 ± 0.5	3.16	0.69	-	0.958	Li [246]
fct FePt/C	8.0 ± 0.5	-	0.7	-	0.945	Li [242]
fct FePt/C	6.5	2.3	1.6	-	-	Chung [244]
fct FePt/C	~6.1	0.589	0.230	-	-	Chen [118]
fct FePt	~3.6	0.37	-	65.8	0.893	Du [250]
fct FePt/CNT	3–13	0.26	0.308	-	0.921	Liu [248]
**CoPt**						
fcc CoPt	8.9 ± 0.8	0.70	0.15	86	-	Li [243]
fcc CoPt/C	2.5 ± 0.2 ^b^	0.57	0.25	-	-	Loukrakpam [119]
fcc CoPt/Co@NHPCC	-	0.876	0.566	-	0.883	Ying [249]
fct PtCo	3.8 ± 1.1	-	0.25 ± 0.07	88	-	Oezaslan [121]
fct CoPt	8.9 ± 0.8	8.26	2.26	66	0.967	Li [243]
**NiPt**						
fcc NiPt/C	4.8 ± 0.5 ^b^	0.69	0.17	-	-	Loukrakpam [119]
fcc NiPt/C	6.1	2.977	0.68	-	-	Carpenter [123]

^a^ Measured at 1.0 V (vs. Ag/AgCl); ^b^ 0.5 M H_2_SO_4_ as electrolyte; ^c^ 1 M of HClO_4_ as electrolyte.

**Table 4:** Available experimental data on MPt (M = Fe, Co) nanoparticles (NPs). M_x_Pt_1−x_ indicates the composition of the system. Structural parameters include size (NP_size_, in nm), shape of the NPs, *c*/*a* ratio and the ordered parameter (S) of NPs. Magnetic proprieties considered in the table include the magnetic state (FM = ferromagnetic, SP = superparamagnetic), the saturation magnetization (M_S_, in emu/cm^3^), the coercivity (H_c_, in Oe), the uniaxial magnetocrystalline anisotropy energy constant (K_u_, in erg/cm^3^), the blocking temperature for the superparamagnetic state (T_B_, in K) and the maximum energy product ((BH)_max_ in MGOe). Magnetic properties are recorded at r.t. and reported in the centimeter/gram/second system (CGS).

MPt	Preparation Method	M_x_Pt_1−x_	Structural Parameters	Magnetic Properties	Reference
**FePt**					
fcc	DC sputtering (p_prep_ = 1 mbar)	Fe_62_Pt_38_	NP_size_ = 4.6, Polycrystalline	H_c_ = 1.48·10^3^, T_B_ = 53–100	Rellinghaus [105]
fcc	Microwave heating method	-	NP_size_ = 2.7	SP	Nguyen [311]
fcc	Synthetic chemical method	-	NP_size_ = 2.25	SP, T_B_ = 14	Nguyen [104]
fcc	Co-reduction chemical method	-	NP_size_~3	SP	Medwal [312]
fcc	Modified polyol process	Fe_52_Pt_48_	-	SP, T_B_ = 20–30	Sun [313]
fcc	Chemical solution method	Fe_52_Pt_48_	NP_size_ = 4	-	Rong [314]
fct Partially Ordered	Microwave heating method + annealing under Ar + 5%H_2_ flowing atmosphere at ~637 K for ~6 min	Fe_48_Pt_52_ (fct > fcc)	NP_size_~24 *c*/*a* = 0.9675	H_c_ = 10.6·10^3^	Nguyen [311]
fct Partially Ordered	DC sputtering (p_prep_ = 1 mbar) + gas-phase sintering at 1073 K	Fe_62_Pt_38_	NP_size_~7.5	T_B_ = 309	Rellinghaus [105]
fct Partially Ordered	DC sputtering (p_prep_ = 1.5 mbar) + gas-phase sintering at 1273 K	Fe_51_Pt_49_	NP_size_~7.2	T_B_ = 309	Rellinghaus [105]
fct Partially Ordered	DC sputtering (p_prep_ = 1 mbar) + gas-phase sintering at 1273 K	Fe_62_Pt_38_	NP_size_~7.7	T_B_ = 530, H_c_ = 1.2·10^3^	Rellinghaus [105]
fct Partially Ordered	Synthetic chemical method + annealing under Ar + 5%H_2_ flowing atmosphere at 662 K for 18 h	Fe_56_Pt_44_ %fcc > %fct	NP_size_ = 6.09	H_c_ = 1.3·10^3^	Nguyen [104]
fct Partially Ordered	Co-reduction chemical method + annealing at 873 K	38% fcc +62% fct	NP_size_~5 *c*/*a* = 0.9848	H_c_ soft-phase = 890 + H_c_ hard-phase = 11,930	Medwal [312]
fct Partially Ordered	Co-reduction chemical method + annealing at 973 K	10% fcc +90% fct	NP_size_~5 *c*/*a* = 0.9801	H_c_ soft-phase = 3250 + H_c_ hard-phase = 12,310	Medwal [312]
fct Partially Ordered	Co-reduction chemical method + annealing at 1023 K	5% fcc + 95% fct	NP_size_ > 5 *c*/*a* = 0.9692	H_c_ soft-phase = 6970 + H_c_ hard-phase = 13,940	Medwal [312]
fct Partially Ordered	Chemical synthesis + annealing at 973 K for 2 h under atmosphere of 4%H_2_	Fe_52_Pt_48_ 24% fcc +76% fct	NP_size_~20 *c*/*a* = 0.9626 S = 0.64	H_c_ = 7212, (BH)_max_~6.31 M_s_ = 34.90	Srivastava [315]
fct	Chemical synthesis + annealing at 973 K for 4 h under 4% H_2_ atmosphere	Fe_52_Pt_48_ 11%fcc + 89%fct	NP_size_~20 *c*/a = 0.9646 S = 0.88	H_c_ = 8617, (BH)_max_~10.92 M_s_ = 30.80	Srivastava [315]
fct	Chemical synthesis + annealing at 973 K for 6 h under atmosphere of 4% H_2_	Fe_52_Pt_48_ 9% fcc + 91% fct	NP_size_~20 *c*/*a* = 0.9626 S = 0.95	H_c_ = 9040, (BH)_max_~7.60 Ms = 32.45, K_u_~6.02·10^7^	Srivastava [315]
fct	Chemical solution route + 1 h annealing under forming gas (Ar + 7% H_2_) at 973 K	Fe_55_Pt_45_	NP_size_ = 10–20	H_c_ = 18·10^3^	Rong [106]
fct	Chemical solution route + 1 h annealing under forming gas (Ar + 7% H_2_) at 973 K	Fe_66_Pt_34_	NP_size_ = 10–20	H_c_ = 7.6·10^3^, (BH)_max_~17	Rong [106]
fct	Chemical solution route + rapid thermal annealing at 923 K for 10 s	Close to Fe_50_Pt_50_	NP_size_ = 8 S = 0.97	(BH)_max_~12.7	Yano [316]
fct	Modified polyol process + annealing at 833 K for 30 min in static N_2_ atmosphere (*p* = 1 atm)	Fe_52_Pt_48_	NP_size_ = 4	FM	Sun [313]
fct	Chemical solution method + annealing at 973 K for 4 h under forming gas (93%Ar + 7%H_2_)	Fe_52_Pt_48_	NP_size_ = 8.2 S~0.93	FM	Rong [314]
**CoPt**					
fcc	Polyol process	Close to Co_50_Pt_50_	NP_size_ = 4 ± 1	H_c_ = 380, M_s_ = 8	Chinnasamy [317]
fcc	Redox transmetallation reaction	Co_46_Pt_54_	NP_size_ = 1.9 ± 0.3	SP, T_B_ = 15	Park [318]
fcc	Soft chemical processing route + annealing at 673 K for 3 h under Ar atmosphere	Co_46_Pt_54_	NP_size_ = 4–7 elongated shape *c*/*a* = 0.9732	H_c_ = 260	Fang [319]
fcc	Redox transmetallation reaction	-	NP_size_~5 with Pt-shell~1.5 nm	H_c_ = 0, T_B_ = 66 M_s_ = 27(//) and 27(⊥)	Bigot [320]
fct	Chemical process + annealing at 923 K for 1 h under Ar/5% H_2_ flowing atmosphere	Co_50_Pt_50_	NP_size_ = 7.6 rod-like shape	H_c_ = 12·10^3^, K_u_ = 1.7·10^7^	Sun [127]
fct	Polyol process + annealing at 823 K for 1 h under H_2_/N_2_ atmosphere	Close to Co_50_Pt_50_	NP_size_ = 4 ± 1	H_c_ = 1.34·10^3^	Chinnasamy [317]
fct	Polyol process + annealing at 873 K or 1 h under H_2_/N_2_ atmosphere	Close to Co_50_Pt_50_	NP_size_ = 4 ± 1	H_c_ = 3.67·10^3^	Chinnasamy [317]
fct	Polyol process + annealing at 973 K for 1 h under H_2_/N_2_ atmosphere	Close to Co_50_Pt_50_	NP_size_ = 4 ± 1	H_c_ = 7.57·10^3^	Chinnasamy [317]
fct	Pellet of NPs obtain from fcc core–shell with a Co-core and a Pt-shell after annealing at 723 K for 1 h in primary vacuum	-	NP_size_ = 8	H_c_ = 50(//) and 50(⊥), T_B_ = 347 M_s_ = 181(//) and 151(⊥) K_u_ ≈ 1.8·10^5^	Bigot [320]

**Table 5:** Experimental preparation methods, size, composition, catalytic performance and magnetic properties of some MPt nanoparticles (M = Fe, Co, Ni) employed as ORR catalysts and reported also in Table 3. The size of the NPs is reported in nm. Catalytic performances are represented by the specific activity (SA, mA·cm−^2^) and the Tafel slope (mV·dec^−1^) measured in a 0.1 M HClO_4_ solution at r.t. and at 0.9 V vs. RHE (unless otherwise specified). Magnetic properties are indicated in the centimeter/gram/second system (CGS) and include magnetic state (FM = ferromagnetic, SP = superparamagnetic) and coercivity (H_c_, Oe) recorded at room temperature.

System	Preparation Method	Size	Composition	SA	Tafel Slope	Magnetic Properties	Reference
**FePt**							
fcc FePt/C	Chemical synthesis	8.5 ± 0.5	As-prepared: Fe_51_Pt_49_; Core–shell: Fe_26_Pt_74_ with a Pt-shell of ~3 atomic layers (~0.6 nm)	0.89 ^a^	-	-	Zhang [247]
fcc FePt/C	Chemical method	<10	Fe_52_Pt_48_	-	-	SP	Li [246]
fcc FePt/C	Bönnemann colloidal method	2.6	~Fe_50_Pt_50_	3.95 ^b^	-	-	Gong [251]
fcc FePt/CNT	Chemical reduction method	2–3	-	-	-	-	Liu [248]
fct FePt/C	Impregnation method + annealing at ~873 K for 3 h under an 8% H_2_/Ar gas mixture	~6.1	Core–shell with ~0.6 nm of Pt coating (2–4 atomic layers)	0.578	-	-	Chen [118]
fct FePt/C	Chemical synthesis + annealing at ~923 K for 1 h under 95% Ar + 5% H_2_ atmosphere	8.5 ± 0.5	As-prepared: Fe_51_Pt_49_; Core–shell: Fe_26_Pt_74_ with a Pt-shell of ~3 atomic layers (~0.6 nm)	2.1 ^a^	-	-	Zhang [247]
fct FePt/C	Chemical method + annealing at ~973 K for 6 h under Ar + 5% H_2_	8.8 ± 0.5	As-prepared: Fe_52_Pt_48_; Core–shell: Fe_50_Pt_50_ with a Pt-shell of ~0.6 nm (~2–4 atomic layers)	3.16	-	H_c_ = 33·10^3^ for as-prepared NPs	Li [246]
fct FePt/C	Modified chemical method + annealing at ~973 K for 6 h under 95% Ar + 5% H_2_ atmosphere	8.0 ± 0.5	Core–shell: Fe_42_Pt_58_ with a Pt-shell of 0.53 nm (~2 atomic layers); degree of ordering >80%	-	-	FM; H_c_ = 33.8·10^3^	Li [242]
fct FePt/C	Chemical synthesis + annealing at ~973 K	6.5	Core–shell with ~0.43 nm of N-doped carbon shell (~2 atomic layers)	2.3	-	-	Chung [244]
fct FePt/C	Impregnation method + annealing at ~1073 K for 3 h under an 8% H_2_/Ar gas mixture	~6.1	Core–shell with ~0.6 nm of Pt coating (2–4 atomic layers)	0.589	-	-	Chen [118]
fct FePt/C	Liquid-phase reduction method + annealing at ~1173 K in a tube furnace under vacuum	~3.6	-	0.37	65.8	-	Du [250]
fct FePt/CNT	Chemical reduction method + annealing at ~923 K under H_2_-free inert atmosphere	3–13	Core–shell with ~3 atomic layers of Pt coating	0.26	-	-	Liu [248]
**CoPt**							
fcc CoPt/C	Chemical method	8.9 ± 0.8	As-prepared: Co_49_Pt_51_	0.70	86	SP	Li [243]
fcc CoPt/C	Chemical synthesis	2.5 ± 0.2	Pt_52_Co_48_	0.57 ^c^	-	-	Loukrakpam [119]
fcc CoPt/ Co@NHPCC	Chemical synthesis	-	-	0.876	-	-	Ying [249]
fct PtCo/C	Liquid precursor impregnation–freeze-drying method + annealing at ~1037 K for 7 h under 4 Vol% H_2_/96 Vol% Ar atmosphere	3.8 ± 1.1	As-prepared: Pt_59_Co_41_ After stability treatment: Pt_77_Co_23_ 85% fct + 15% fcc	-	88	-	Oezaslan [121]
fct CoPt/C	Chemical method + annealing at ~923 K for 6 h under 95% Ar + 5% H_2_ atmosphere	8.9 ± 0.8	As-prepared: Co_49_Pt_51_; Core–shell with a Pt-shell of 3 atomic layers 88% fct + 12% fcc	8.26	66	FM; H_C_ = 7.1·10^3^ for as-prepared NPs	Li [243]
NiPt							
fcc NiPt/C	Chemical synthesis	4.8 ± 0.5	Pt_56_Ni_44_	0.69 ^c^	-	-	Loukrakpam [119]
fcc NiPt/C	Solvothermal reaction	6.1	Ni_47_Pt_53_	2.977	-	-	Carpenter [123]

^a^ Measured at 1.0 V (vs. Ag/AgCl); ^b^ 1 M of HClO_4_ as electrolyte; ^c^ 0.5 M H_2_SO_4_ as electrolyte.

**Table 6:** Application of external magnetic field directly or indirectly, through the magnetization of the catalyst, for ORR catalysts. MF and FM stand for magnetic field and ferromagnetic, respectively.

Catalyst	Three-Electrode and External Magnetic Field Parameters	Experimental Observations	Ref.
**Direct application of external magnetic fields**			
Pt/ FM CoPt nanowire/alumina membrane	Rotating disk working electrode Pt plate as counter-electrode Reference electrode: Ag/AgCl O_2_-/air-saturated buffer electrolyte solution (pH = 8.4 and T = 298 K) MF of H~0.4, 1 T is generated by large electromagnet (200 mm pole faces) FM nanostructures are placed behind Pt layer of the working electrode to achieve large MF gradient	FM CoPt nanowires behind Pt layer and external MF increase ORR current by almost an order of magnitude in comparison with Pt working electrode and in MF absence FM-CoPt-containing electrode in MF ~1 T leads to average current enhancement of 118% in polarization experiments and 297% in chronoamperometry Considerable ORR enhancement is obtained by combing effects of applied MF and effects of the intrinsic ferromagnetism of CoPt nanowires The synergy between applied MF and magnetized electrode attracts paramagnetic species, such as${\;}^{3}O_{2}$ and ${\mathrm{HO}}_{2}^{-}$, to the electrode surface and repels diamagnetic ones, such as H_2_O	Chaure [377]
Pt/alumina membrane			
**Indirect application of external magnetic fields (magnetization of catalyst)**			
L1_0_-PtFe nanopillar (FM)	Working electrode: L1_0_-FePt + Ag paste + Cu wire coated in glass Pt foil as counter-electrode Reference electrode: Ag/AgCl O_2_-saturated 0.1 M KOH electrolyte solution MF of H = ±7 T is applied along the normal direction of the film L1_0_-PtFe exhibits typical anisotropic out-of-plane after MF application	Current density is five times higher with magnetized L1_0_-PtFe NF than with non-magnetized catalyst MF generated by magnetized catalyst affects coverage of chemisorbed oxygen Manipulation of O-coverage at the catalysts by MF is key aspect in ORR regulation MF realigns the spins of the whole catalyst in a single direction	Lu [379]
Pt/Ag/CoPt nanowire	Working electrode: Pt/Ag/CoPt nanowire embedded in alumina Pt as counter-electrode Reference electrode: Ag/AgCl O_2_-/N_2_-saturated buffer electrolyte solution (pH = 8.4 and T = 298 K) MF gradient is produced with H ~20–25 mT at Pt surface CoPt nanowires are magnetized along their lengths (// to each other)	200% enhancement of reaction current is obtained with stationary magnetized electrode Enhancement in reaction current is reported when magnetized electrode is spinning MF at electrode surface is too small to significantly improve the equilibrium concentration of ^3^O_2_ at surface ORR is enhanced by ~10–20% in oxygenated alkaline medium	Chaure [378]

**Table 7:** Descriptions, advantages, limitations and examples for all types of electrochemical energy storage systems.

Battery	Characteristics	Advantages	Limitations	Examples
**Primary** **batteries**	single-use design aqueous and non-aqueous types used in portable devices	convenient and cheap simple and ready to use high abundance of raw materials	not fully electrically rechargeable corrosion problems environmentally unfriendly	Zinc–carbon, alkaline, lithium primary cells
**Secondary batteries**	rechargeable wide day-to-day applications (e.g., portable devices and car ignition for hybrid vehicles)	many charge/discharge cycles high abundance of raw materials high recyclability	high costs of some metal components short life cycle corrosion problems	Lead acid, lithium ion, nickel–cadmium
**Battery** **systems for grid-scale** **energy**	integrated with smart intelligent grid provide large amounts of high-quality power quickly and for a long period	large-scale storage systems	high cost toxicity of some metals used as raw materials	Flow, sodium–sulfur
**Fuel cells**	continuous conversion of chemical into electrical energy split into direct and indirect systems	efficient energy conversion flexible scaling reliable power environmentally friendly wide range of applications	high cost of materials (rare noble metals) difficult fuel production, storage and transportation complicated design stability and durability issues	Proton exchange membrane, direct methanol, solid oxide
**Electrochem. capacitors** **(or supercapacitors)**	energy stored in an electric double layer under an applied voltage use in small devices	low charge times long cell life high specific power	efficiency issues	Carbon-based, metal oxide, polymeric

**Table 8:** SWOT analysis of fuel cells.

Strength	Weakness	Opportunities	Threats
high thermodynamic efficiency (40–60%) co-generation of electricity and heat flexible electrical power production (50 W–100 MW) constant high efficiency for small-scale units and under full/partial load conditions minor environmental issues (negligible pollution when H_2_ is used as main fuel) quiet system	durability and stability issues catalytic lifetime high investment costs (expensive raw materials)	market penetration (stationary and portable applications) domestic and industrial heat and power source medical applications integration with intermittent renewable energy sources non-road applications as auxiliary power units (APU) for aviation, maritime, rail and off-road sectors applications as backup power in telecoms	production, storage, transport of hydrogen and related safety issues expensive and time-consuming controlling procedures for many units raw materials limits maturating battery technologies

**Table 9:** PEMFC parameters and experimental observations in applied experiments carried out in the presence of an external magnetic field or magnetic catalysts. MF and RH stand for magnetic field and relative humidity, respectively.

Catalyst (Cathode)	PEMFC and MF Parameters	Experimental Observations	Reference
**Single Fuel Cell experiments under an applied magnetic field**			
Pt	Carbon paper with Pt as gas diffusion layer, Nafion 117 (PEM) and bipolar acrylic plates H_2_/O_2_ (2:1) as fuel (flow rate ~30 mL/min, partial P_O2_~0.1 atm and T = 281 K) Fe-Nd-B magnets outside PEMFC cell and MF ⊥ to electrodes	MF can improve cell voltage at high current density depending on MF gradient direction (when O_2(g)_ transport is limited by diffusion) MF affects more O_2_ transport and mass transfer through gas diffusion area to catalytic surface than the catalysis in itself	Matsushima [397]
Pt	PEMFC effective area ~4.84 cm^2^ Inlet humidity at the electrodes = 100% RH Back pressure at the electrodes = 0.1 mPa and T = 333 K Magnets at the anode side MF ~100, 200, 300, 400 mT	Improved electric efficiency Inhibition of the degradation of the battery performance Extension of the PEMFC service life Facilitate the removal of water molecules Reduction in H_2_ penetration through the PEM to the anode	Lang [419]
Not reported	Graphite flow field plates for gas distribution Cu plates as current collectors Graphite plate as electrodes H_2_/air (2.0:1.5) as fuel (T = 343 K) Fe-Nd-B magnets outside PEMFC cell and MF ⊥ to electrodes MF ~100, 300 and 500 mT	~27% improvement of peak power density when MF is applied at B = 500 mT Overall performance increases with the increment in MF strength ΔTemp between cathode and anode increases with MF strength increment RH decreases when MF is applied (improved removal of water)	Ruksawong [420]
**Single Fuel Cell experiments with magnetized catalyst**			
Nd-Fe-B + 20% Pt/Vulcan XC72	Nd-Fe-B+Pt/C+20 wt % as cathode and Pt/C as anode (Pt loading = 0.5 mg/cm^2^ at anode and cathode) H_2_/air as fuel (T = 353 K) H ~4 T for magnetizing either cathode or MEA MF ⊥ to cathode electrode	Increased efficiency in magnetized compared to non-magnetized MEA Simulations indicate that catalytic permanent magnets play a role in water management and enhancement of the oxygen transport	Okada [381]
Pt-Co/ MWCNTs	MEA fabricated by sandwiching Nafion 212 membrane between anode and cathode (Pt loading ~0.4 mg/cm^2^ at anode and cathode) C papers as diffusion layers H_2_/air as fuel (RH ~80%, T = 333 K) H = 350 mT for magnetizing the electrodes	Magnetized MEA improves peak output power density by 49.5% (vs. non-magnetized) MF application produces an increment of 29.6% of the electrochemical surface area Reaction resistance of the magnetized MEA is lower than non-magnetized one	Sun [421]
Nd_2_Fe_14_B/C+ 50% Pt/C	MEA includes polyester frame, anode, Nafion 1135 film, cathode, polyester frame (Pt/C load density = 0.80 mg/cm^2^ at the cathode and anode; Nd_2_Fe_14_B/C load density = 0.40, 0.80 mg/cm^2^) Carbon paper as diffusion layer H_2_/air as fuel (pressure = 0.11 MPa) and T = 343 K H~350 mT for loading the catalyst on the working electrode H~2 T for magnetizing the electrode	MF increases the conductivity at cathode and accelerates oxygen mass transfer Discharge current increases by ~40% at 0.20 V voltage under MF Better discharge performance is obtained when magnetized Nd_2_Fe_14_B/C load density is 0.40 mg/cm^2^ instead of 0.80 mg/cm^2^ Decrement in performance at higher magnetized load density Cathode ohmic polarization is decreased by Nd_2_Fe_14_B/C particles Oxygen transfer is promoted by MF	Shi [384]
**Fuel cell stack experiments**			
Pt	Carbon paper with Pt as gas diffusion layer H_2_/air gasses as fuel (H_2(g)_ inlet *p* = 0.7 bars, air at 40 RH, T = 323 K) MF is generated by Cu electromagnetic coil and fuel cell is placed in the middle MF ~16 and 26 mT MF without a specific direction	Increment in the electricity production with MF Weak MF remarkably affects performance provided enough air is supplied Output volt increases with MF strength Enhancement of ~10% efficiency not affected by MF direction	Abdel-Rehim [418]

**Table 10:** Reported experimental effects in electrochemical processes and devices such as PEM fuel cells, when magnetic catalysts containing FM metals ($\vec{H_{0}}$) are employed, when an external magnetic field is applied ($\vec{H}$) and when the two effects are combined ($\vec{H_{0}}+\vec{H}$).

Effects of Engineering Magnetic Catalysts with FM Metals $\left(\vec{H_{0}}\right)$	Effects of Applying an External Magnetic Field $\left(\vec{H}\right)$	Effects of Combining Engineered Magnetic Catalysts with FM Metals and Applied External Magnetic Field $\left(\vec{H_{0}}\;+\;\vec{H}\right)$
Lower activation barriers Spin selection Improved structural stability of the catalyst Improved conductivity	improved mass transport and electron transfer mass transfer rate of gasses such as ^3^O_2_ and H_2_ activation of reagents such as ^3^O_2_ and H_2_ spin modulation on FM materials modulation of the electrochemical double layer (EDL) stabilization of the electrochemical membrane	enhanced overall efficiency of the electrochemical cell improved stability and durability of MEA

## Data Availability

Not applicable.
